# Synthesis and selected transformations of 2-unsubstituted 1-(adamantyloxy)imidazole 3-oxides: straightforward access to non-symmetric 1,3-dialkoxyimidazolium salts

**DOI:** 10.3762/bjoc.15.43

**Published:** 2019-02-19

**Authors:** Grzegorz Mlostoń, Małgorzata Celeda, Katarzyna Urbaniak, Marcin Jasiński, Vladyslav Bakhonsky, Peter R Schreiner, Heinz Heimgartner

**Affiliations:** 1 Department of Organic and Applied Chemistry, University of Łódź, Tamka 12, PL-91-403 Łódź, Poland; 2 Justus Liebig University, Institute of Organic Chemistry, Heinrich-Buff-Ring 17, D-35392 Giessen, Germany; 3 Department of Chemistry, University of Zurich, Winterthurerstrasse 190, CH-8057 Zurich, Switzerland

**Keywords:** alkoxyamines, imidazole *N*-oxides, imidazolium salts, nucleophilic carbenes, sulfur transfer reaction

## Abstract

Adamantyloxyamine reacts with formaldehyde to give *N*-(adamantyloxy)formaldimine as a room-temperature-stable compound that exists in solution in monomeric form. This product was used for reactions with α-hydroxyiminoketones leading to a new class of 2-unsubstituted imidazole 3-oxides bearing the adamantyloxy substituent at N(1). Their reactions with 2,2,4,4-tetramethylcyclobutane-1,3-dithione or with acetic acid anhydride occurred analogously to those of 1-alkylimidazole 3-oxides to give imidazol-2-thiones and imidazol-2-ones, respectively. Treatment of 1-(adamantyloxy)imidazole 3-oxides with Raney-Ni afforded the corresponding imidazole derivatives without cleavage of the N(1)–O bond. Finally, the *O*-alkylation reactions of the new imidazole *N*-oxides with 1-bromopentane or 1-bromododecane open access to diversely substituted, non-symmetric 1,3-dialkoxyimidazolium salts. Adamantyloxyamine reacts with glyoxal and formaldehyde in the presence of hydrobromic acid yielding symmetric 1,3-di(adamantyloxy)-1*H*-imidazolium bromide in good yield. Deprotonation of the latter with triethylamine in the presence of elemental sulfur allows the in situ generation of the corresponding imidazol-2-ylidene, which traps elemental sulfur yielding a 1,3-dihydro-2*H*-imidazole-2-thione as the final product.

## Introduction

**Scheme 1:**
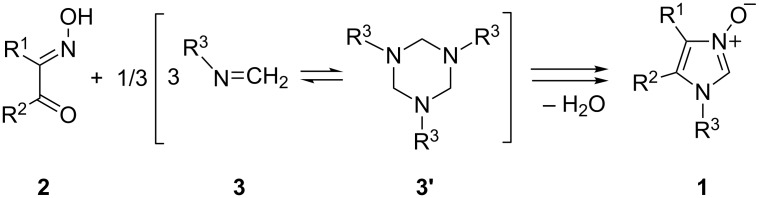
Synthesis of 2-unsubstituted imidazole *N*-oxides **1** from α-hydroxyiminoketones **2** and formaldimines **3**.

Imidazole *N*-oxides constitute a practically valuable class of five-membered aromatic *N*-heterocycles [1–5]. The subclass of 2-unsubstituted imidazole *N*-oxides **1** with diverse substituents located at N(1), C(4), and C(5) is of special interest as so-called ‘nitrone like’ reagents for the synthesis of more complex, imidazole containing systems (Scheme 1) [6]. These imidazole *N*-oxides are easily accessible via heterocyclization reactions comprising condensation of α-hydroxyiminoketones **2** with formaldimines **3**. The latter are known to exist in monomeric form in the case of sterically crowded parent amines such as 1-aminoadamantane or *tert*-butylamine or, alternatively, as trimeric hexahydro-1,3,5-triazines **3’** in the case of sterically less crowded amines [7]. In analogy to other azoles and in contrast to six-membered aromatic *N*-heterocycles, *N*-oxides **1** cannot be prepared via oxidation of the parent imidazoles by treatment with an oxidizing agent, e.g., with a percarboxylic acid [6].

Imidazole *N*-oxides are versatile substrates for the preparation of diverse imidazole derivatives and the most characteristic feature is their 1,3-dipolar reactivity analogous to aldonitrones, which enables their conversion via [3 + 2]-cycloadditions with dipolarophiles such as activated ethylenes [8–9], activated acetylenes [10–11] or isocyanates [11–12]. The initially formed [3 + 2]-cycloadducts undergo spontaneous secondary conversions leading to re-aromatization of the imidazole ring. The same mechanism governs sulfur-transfer reactions with cycloaliphatic thioketones yielding the corresponding imidazole-2-thiones [13]. The isomerization of *N*-oxides **1** into the corresponding imidazol-2-ones can be easily performed by treatment with acetic anhydride at room temperature [14]. An important reaction of **1** is the *O*-alkylation leading to alkoxyimidazolium salts, which display in some cases properties of ‘room temperature ionic liquids’ [15–16]. Finally, straightforward deoxygenation by treatment with Raney-Ni is also worth mentioning [17].

Condensations presented in Scheme 1 occur smoothly with formaldimines **3** derived from aliphatic amines, but in the case of primary aromatic amines harsher reaction conditions are required [6]. To date, no synthesis of 1-alkoxyimidazole *N*-oxides derived from alkoxyamines (‘hydroxyamine ethers’) has been reported. An alternative approach to these products comprises the alkylation (in most cases methylation) of 1-hydroxyimidazole *N*-oxides. However, the products of the monomethylation could not be obtained, but symmetric *N,N*-disubstituted imidazolium salts resulting from double *O*-alkylation were isolated [18].

On the other hand, alkoxyamines are known not only as bioactive compounds [19–20] but also as important initiators of polymerization processes that have been extensively studied in the recent decade [21]. Among alkoxyamines, adamantyloxyamine (**4**) occupies a prominent position, and for that reason it was selected for experiments aimed at the preparation of the new group of 2-unsubstituted imidazole *N*-oxides bearing the adamantyloxy residue at N(1). The goal of the present study was the synthesis of some representatives of this type and comparison of their properties with those of 1-adamantyl analogues.

## Results and Discussion

**Scheme 2:**
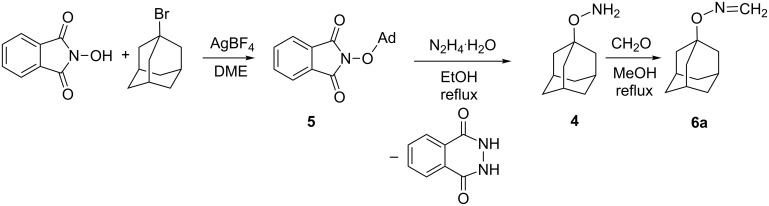
Preparation of adamantyloxyamine (**4**) and its conversion into *N*-(adamantyloxy)formaldimine (**6a**); Ad = 1-adamantyl.

The starting adamantyloxyamine (**4**) was prepared from 1-bromoadamantane, which in the presence of an equimolar amount of AgBF_4_ in boiling dimethoxyethane (DME) reacts with *N*-hydroxyphthalimide to give the *O*-alkylation product **5** [20] (Scheme 2). The latter was converted to **4**, obtained in 86% yield, after hydrazinolysis with N_2_H_4_**^.^**H_2_O in slight excess. The crude product was reacted with formaldehyde in boiling MeOH, and after 1 h, crystalline *N*-(adamantyloxy)formaldimine (**6a**) was isolated in 90% yield. The spectroscopic data indicate that this imine exists in solution in monomeric form exclusively. Thus, the ^1^H NMR spectra confirmed this structure by the presence of two doublets located at 6.39 and 7.01 ppm with *J* = 12 Hz, characteristic for the =CH_2_ group. In addition, the ^13^C NMR spectrum showed the absorption of this group at 136.1 ppm.

**Scheme 3:**
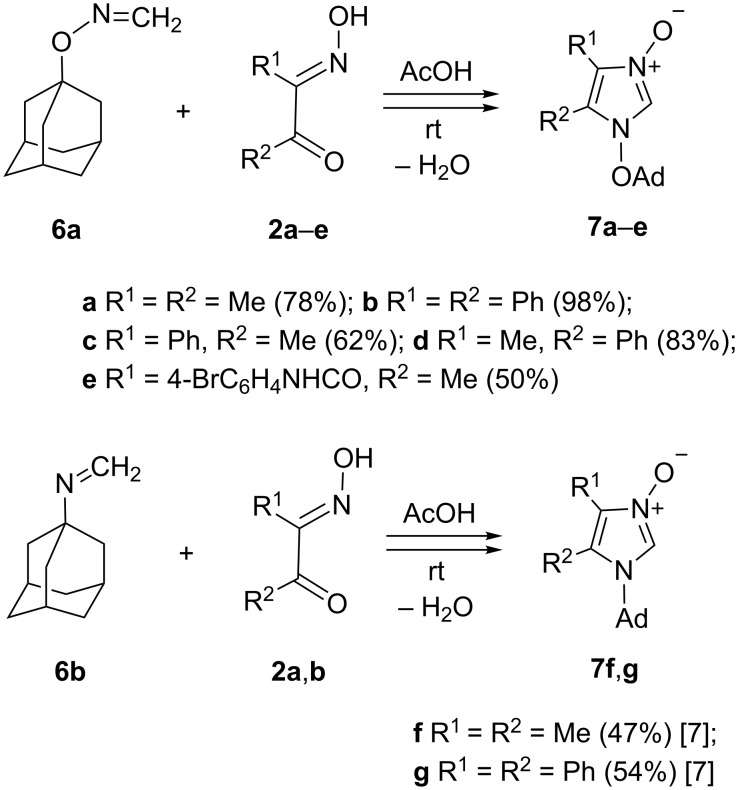
Synthesis of 1-(adamantyloxy)imidazole 3-oxides **7a**–**e** and 1-adamantylimidazole 3-oxides **7f**,**g** in acetic acid at room temperature.

Having imine **6a** in hands, syntheses of a series of 2-unsubstituted 1-(adamantyloxy)imidazole 3-oxides **7a–e** were performed in glacial acetic acid at room temperature. The crude products were transformed into their hydrochlorides by treatment with conc. hydrochloric acid, and subsequent neutralization over solid NaHCO_3_ led to crystalline products (procedure A). Following this procedure, the required *N*-oxides **7a**–**e** were obtained in good to excellent yields (Scheme 3). In addition, based on the earlier described protocol [7], two imidazole *N*-oxides **7f** and **7g**, bearing the adamantan-1-yl moiety attached to N(1), were also obtained in high yields starting with (adamantyl)formaldimine (**6b**, Scheme 3). In this case, crude products were isolated as hydrochlorides not by treatment with hydrochloric acid but with gaseous hydrogen chloride (procedure B). Both compounds obtained by this method were fully characterized and described in an earlier publication [7].

In all cases the ^1^H NMR spectra of new products confirmed the structures **7a**–**d** by the presence of the diagnostic HC(2) signal between 8.76 and 7.93 ppm. In the ^13^C NMR spectra, absorptions of the three imidazole C-atoms were found between 120 and 130 ppm. In addition, the signals of OC(1) of the adamantyl skeleton appeared in a narrow range at 89.0–86.9 ppm. Unexpectedly, in the case of **7e**, the same procedure led to the final, crystalline product as a hydrochloride. Apparently, hydrogen bonding in this molecule is strong enough to bind an HCl molecule, which cannot be removed by treatment with solid NaHCO_3_ in the methanolic solution under standard conditions.

**Scheme 4:**
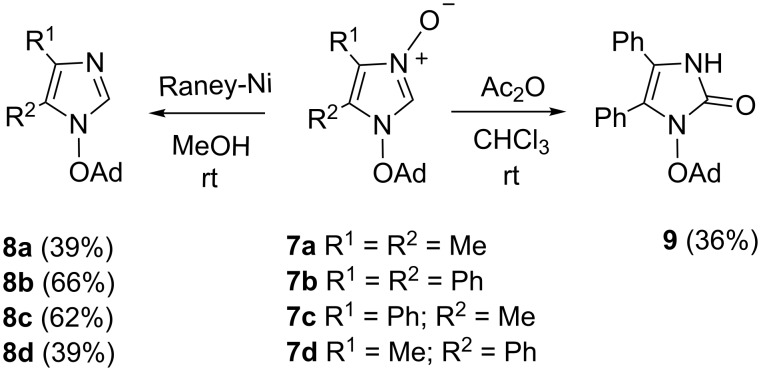
Deoxygenation of 1-(adamantyloxy)imidazole 3-oxides **7a**–**d** and isomerization of **7b** into imidazole-2-one **9**.

The first question was whether the new imidazole *N*-oxides **7** can be deoxygenated with Raney-Ni without cleavage of the N(1)–OAd bond. The test reaction with **7a** demonstrated that the reaction performed in MeOH solution at room temperature was completed after 2 h, and the main product isolated in 39% yield was the expected 1-(adamantyloxy)imidazole **8a** (Scheme 4). In the product, the diagnostic HC(2) signal in the ^1^H NMR spectrum shifts high-field and appears at 7.34 ppm. Analogously, deoxygenation of *N*-oxides **7b–d** led to the required imidazoles **8b–d** in 66%, 62%, and 39% yield, respectively.

As pointed out in the introduction, one of the most typical reactions of 2-unsubstituted imidazole *N*-oxides is their isomerization to imidazol-2-ones. In many reported cases, this transformation can be performed by treatment with acetic anhydride, heating in a high boiling solvent or photolytically [6]. In a test experiment with **7b**, the reaction with Ac_2_O in CHCl_3_ solution at room temperature overnight led to a crystalline product identified as the expected imidazole-2-one **9** in 36% yield (Scheme 4). In that case, the ^1^H NMR singlet located at 11.02 ppm belongs to HN(3). The ^13^C NMR spectrum confirmed the structure of **9** by the signal of the C(2)=O group found at 153.0 ppm. The corresponding band in the IR spectrum appeared at 1702 cm^−1^as the strongest absorption.

Thermal isomerization of **7b** was also tested. However, boiling in bromobenzene for 15 min resulted in decomposition of the starting material, and in this case, the expected imidazole-2-one **9** could not be detected in the crude reaction mixture.

**Scheme 5:**
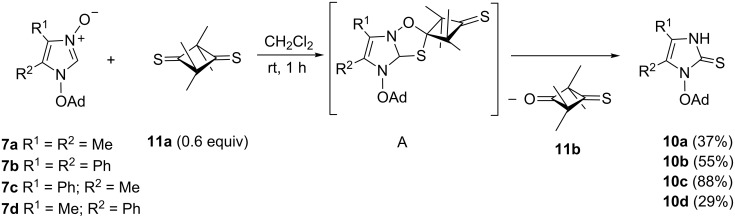
Conversions of imidazole 3-oxides **7a**–**d** into 1-(adamantyloxy)imidazole-2-thiones **10a**–**d** via sulfur transfer reaction.

Sulfur-transfer reactions offer an attractive approach to imidazole-2-thiones starting with 2-unsubstituted imidazole *N*-oxides [13]. The importance of this procedure is reflected in the multistep reactions applied for the preparation of some bioactive imidazole derivatives [22–23]. However, the preparation of imidazole-2-thiones with *N*-alkoxy groups, starting with the corresponding 2-unsubstituted imidazole 3-oxides, has not yet been reported. It turned out that the imidazole *N*-oxide **7b** can smoothly be converted into 1-(adamantyloxy)imidazole-2-thione **10b** in the presence of 2,2,4,4-tetramethylcyclobutane-1,3-dithione (**11a**) as the sulfur-donating reagent. The reaction was carried out in CH_2_Cl_2_ at room temperature, and after 1 h, the desired product **10b** was isolated in 55% yield (Scheme 5); its structure was confirmed spectroscopically. For example, in the ^1^H NMR spectrum a singlet located at 12.15 ppm was attributed to the HN(3) unit. Moreover, the ^13^C NMR spectrum revealed the C=S absorption at 159.6 ppm. Analogous transformations were achieved with **7a** and **7c**,**d**. A plausible explanation of the sulfur-transfer mechanism via the intermediate [3 + 2]-cycloadduct **A** is presented in Scheme 5. The eliminated monothione **11b** enters an analogous reaction with the starting imidazole *N*-oxide **7**, leading to a second molecule of **10** and 2,2,4,4-tetramethylcyclobutane-1,3-dione.

Imidazolium salts are of special importance as they are widely used as ionic liquids or precursors of imidazole-based nucleophilic carbenes (imidazol-2-ylidenes). For example, deprotonation of 1,3-diadamantylimidazolium chloride led to the first stable imidazol-2-ylidene (the so-called ‘Arduengo carbene’) [24–25]. Alkylation of 2-unsubstituted imidazole *N*-oxides is a known procedure for the preparation of alkoxyimidazolium salts [16], and the alkylation of *N*-oxides of type **7** should lead to 1,3-dialkoxyimidazolium salts, which form a little-known class of imidazole derivatives. The method reported earlier for their synthesis comprises the two-fold alkylation (methylation with Me_2_SO_4_ or benzylation using benzyl bromide) of some 1-hydroxyimidazole 3-oxides leading to symmetric 1,3-dialkoxyimidazolium salts [18]. In a recent publication, however, sequential alkylation of 1-hydroxyimidazole 3-oxide using two different alkyl halides (R^1^X and R^2^X; R^1^, R^2^ = Me, Et, *n*-Bu, or allyl) as alkylating agents, leading to non-symmetric 1,3-dialkoxyimidazolium salts, was also reported [26].

**Scheme 6:**
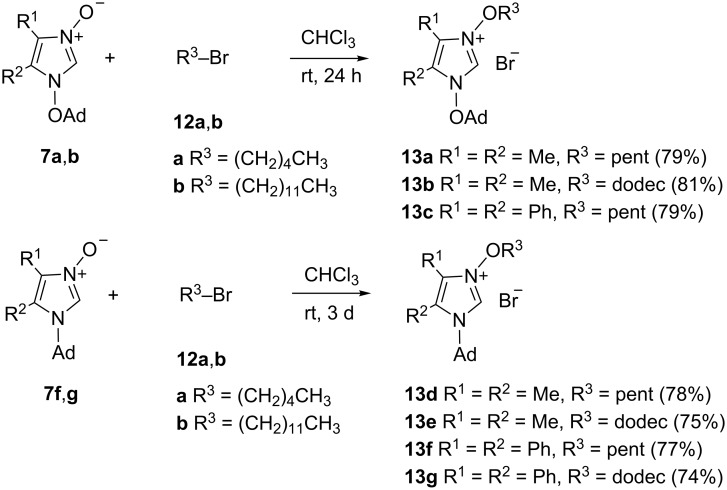
Syntheses of the non-symmetric 1,3-dialkoxyimidazolium bromides **13a**–**c** and 1-alkyl-3-alkoxyimidazolium bromides **13d**–**g**.

In the present study, imidazole *N*-oxides **7** bearing either an adamantyloxy or adamantyl moiety at N(1) were smoothly alkylated with 1-bromopentane (**12a**) or 1-bromododecane (**12b**) in CHCl_3_ at room temperature. In the case of **7a** and **7b**, both reactions were completed after 24 h and a crystalline product was isolated in each case. The NMR spectra confirmed the expected structure of 1-adamantyloxy-3-alkoxyimidazolium bromides **13a** and **13b**, respectively (Scheme 6). In the ^1^H NMR spectra of both compounds, the most characteristic signal of HC(2) was shifted significantly to lower field and appeared at 11.46 and 12.12 ppm, respectively. In the ^13^C NMR spectra of **13a**, two signals at 83.9 and 91.5 ppm were attributed to CH_2_–O and C–O of the pentyloxy and adamantyloxy residues.

In order to compare the course of the alkylation reactions of **7a** and **7b** with those of the structurally analogous 1-adamantylimidazole 3-oxides **7f** and **7g**, the latter were treated with **12a** or **12b** under the same conditions. Remarkably, these alkylations occurred more slowly and their completion was established only after 3 d leading to 1-adamantyl-3-alkoxyimidazolium bromides **13d**–**g**. These results indicate that the 1-adamantyloxy substituent enhances the nucleophilicity of the *N*-oxides and the alkylation requires shorter reaction times.

In extension of this study, the alkylation of 1-adamantyloxy-4,5-diphenylimidazole (**8b**) with **12b** was also attempted under the same conditions. In that case, however, the expected *N*-alkylation did not occur at room temperature and even after 2 d the starting materials were found unchanged in the reaction mixture. On the other hand, the attempted *N*-alkylation of **8b** upon MW irradiation led to the formation of a mixture of starting materials and some unidentified decomposition products.

**Scheme 7:**
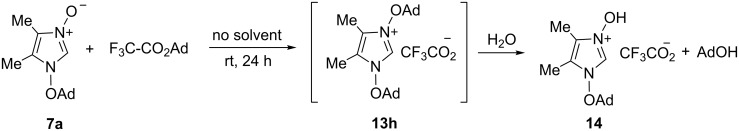
Attempted *O*-adamantylation of imidazole *N*-oxide **7a** with adamantan-1-yl trifluoroacetate and subsequent hydrolysis of the initially formed imidazolium salt **13h**.

Due to the great importance of both carbocyclic and heterocyclic systems functionalized with the adamantyl group [27], different types of adamantylation reactions (*C*-, *N*-, or *O*-adamantylation) attract attention, and typically 1,3-dehydroadamantane [28] or 1-haloadamantanes [29] are applied as alkylating reagents. Adamantan-1-yl carboxylates are also known as efficient adamantylating reagents [30]. In spite of the fact that adamantylations of some benzimidazoles and imidazoles have already been reported [31], similar reactions with heterocyclic *N*-oxides have not been published yet. For that reason, in the final part of the study, a preliminary experiment aimed at the *O*-adamantylation of imidazole *N*-oxide **7a** was carried out under typical conditions (CHCl_3_ solution, rt) using 1-bromoadamantane as an alkylating agent. However, formation of the expected 1,3-bis(adamantyloxy)imidazolium salt was not observed neither in the absence nor in the presence of AgBF_4_. Based on this observation, 1-bromoadamantane was replaced by adamantan-1-yl trifluoroacetate (Scheme 7). Unexpectedly, this test experiment, performed with **7a** at room temperature, led after 24 h not to the expected, symmetric 1,3-di(adamantyloxy)imidazolium salt **13h** but to the trifluoroacetate of the starting material, i.e., compound **14**, formed side by side with adamantan-1-ol. Apparently, the initially formed **13h** underwent spontaneous hydrolysis in the presence of air moisture, leading to the mixture of both isolated products.

**Scheme 8:**
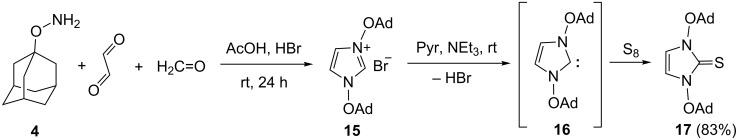
Synthesis of the symmetric 1,3-di(adamantyloxy)imidazolium bromide (**15**) and its transformation to 1,3-dihydro-2*H*-imidazol-2-thione **17** via the intermediate imidazol-2-ylidene **16**.

Finally, in extension of the study focused on the preparation of new, symmetric 1,3-dialkoxyimidazolium salts, the synthesis of 1,3-di(adamantyloxy)imidazolium salt, starting with glyoxal hydrate, adamantyloxyamine (**4**) and formaldehyde, using these reagents in a ratio 1:2:1, in the presence of hydrobromic acid was attempted. The reaction performed overnight in acetic acid at room temperature led to the expected imidazolium bromide **15** in 41% yield (Scheme 8). Its structure was confirmed by spectroscopic data; for example, in the ^1^H NMR spectrum the characteristic singlets of H(2) and H(4)/H(5) appeared at 11.90 and 7.68 ppm, respectively, and the ratio of the intensities was 1:2. On the other hand, the ^13^C NMR revealed the absorptions of C(2) and C(4)/C(5) at 137.0 and 120.2 ppm, respectively.

Unfortunately, attempted syntheses of analogous, symmetric 1,3-dialkoxyimidazolium bromides, derived from diacetyl or 1,2-diphenylethane-1,2-dione, using the same protocol, were unsuccessful.

Nucleophilic carbenes are known to undergo conversion into imidazole-2-thiones by trapping of elemental sulfur [26,32]. Treatment of the symmetric imidazolium salt **15** with triethylamine in pyridine solution in the presence of elemental sulfur led to the 1,3-dihydro-2*H*-imidazole-2-thione **17** isolated in 83% yield (Scheme 8). In the ^1^H NMR spectrum, two equivalent HC(4) and HC(5) atoms appeared at 6.69 ppm, and in the ^13^C NMR spectrum, the C(4) and C(5) atoms gave only one signal localized at 114.6 ppm. Apparently, deprotonation of the imidazolium cation results in the formation of the nucleophilic imidazol-2-ylidene **16**, which in situ reacts with elemental sulfur dissolved in pyridine solution yielding the symmetrically substituted 1,3-dihydro-2*H*-imidazole-2-thione **17**.

## Conclusion

The present study demonstrates that the heterocyclization reaction with α-hydroxyiminoketones and formaldimines leading to 2-unsubstituted imidazole 3-oxides can efficiently be performed with *N*-(adamantyloxy)formaldimine, and 2-unsubstituted 1-(adamantyloxy)imidazole 3-oxides were obtained in high yields. In general, they react similarly to their 1-alkyl analogues and undergo deoxygenation without removal of the adamantyloxy fragment. The sulfur transfer reactions provide access to the new group of 1-(adamantyloxy)imidazole-2-thiones, which potentially are of interest for medicinal chemistry. The acetic acid anhydride assisted isomerization reaction of a new *N*-oxide leads to the corresponding imidazol-2-one. However, the attempted thermal isomerization of a 1-(adamantyloxy)imidazole *N*-oxide resulted in decomposition of the starting material. The alkylation experiments performed with 1-bromopentane and 1-bromododecane showed that 1-(adamantyloxy)imidazole 3-oxides are more reactive than their 1-adamantanyl analogues, and the corresponding 1,3-dialkoxyimidazolium bromides were obtained in high yields. Attempted adamantylation of a 1-(adamantyloxy)imidazole 3-oxide with 1-bromoadamantane or adamantan-1-yl trifluoroacetate was unsuccessful. Nevertheless, the corresponding symmetric 1,3-di(adamantyloxy)imidazolium bromide was obtained in the reaction of adamantyloxyamine with glyoxal, formaldehyde and hydrobromic acid. By treatment with triethylamine, it was deprotonated and formed the corresponding imidazol-2-ylidene, which reacts with elemental sulfur yielding the expected 1,3-dihydro-2*H*-imidazole-2-thione. Thus, the elaborated protocols provide straightforward access to diverse 1-(adamantyloxy)imidazole 3-oxides as well as to 1,3-dialkoxyimidazolium salts, which are attractive substrates for syntheses of other imidazole derivatives, including a new group of 1-alkoxy and 1,3-dialkoxyimidazol-2-ylidenes. In all described cases, it is of interest to probe the influence of the alkoxy residues on the stability and reactivity of the hitherto unknown nucleophilic carbenes bearing this groups at N(1) and/or N(3) atoms.

In addition, it is worth mentioning that 1,3-dibenzyl-4,5-dimethylimidazolium chloride as well as its 2-methyl-substituted analogue are well known imidazole alkaloids, which found wide application in some regions as food-stuff and medical supply [33]. The method described in the present study opens a straightforward access to their benzyloxy analogues, potentially bioactive compounds, which have not been known yet.

## Experimental

**General information:** Solvents and chemicals were purchased and used as received without further purification. Products were purified by standard column chromatography on silica gel (230–400 mesh, Merck). Unless stated otherwise, yields refer to analytically pure samples. NMR spectra were recorded with a Bruker Avance III 600 MHz instrument (^1^H NMR: 600 MHz; ^13^C NMR: 151 MHz). Chemical shifts are reported relative to solvent residual peaks (^1^H NMR: δ = 7.26 ppm [CHCl_3_]; ^13^C NMR: δ = 77.0 ppm [CDCl_3_]). IR spectra were registered with a FTIR NEXUS spectrometer (as film or KBr pellets). Melting points were determined in capillaries with a Stuart SMP30 apparatus with automatic temperature monitoring.

**Starting materials:** Adamantyloxyamine (**4**) was prepared following a modified published procedure [20]. For the first step, the *O*-adamantylation of *N*-hydroxyphthalimide, the recommended diethyl ether as a solvent was replaced by 1,2-dimethoxyethane. Methylideneadamantylamine (**6b**) was prepared by treatment of 1-aminoadamantane, isolated from commercial 1-aminoadamantane hydrochloride after neutralization of its aqueous solution with diluted sodium hydroxide solution, with paraformaldehyde [34]. 1-(Adamantan-1-yl)-4,5-dimethylimidazole *N*-oxide (**7f**) and 1-(adamantan-1-yl)-4,5-diphenylimidazole *N*-oxide (**7g**) were obtained as crystalline materials following a published protocol (method B) [7]. α-Hydroxyiminoketones **2a**,**c**–**e** were prepared based on the earlier described procedure via nitrosylation of the corresponding ketones or β-ketoamides with isoamyl nitrate [35] or, as in the case of **2b,** by oximation of 1,2-diphenylethane-1,2-dione [36]. 2,2,4,4-Tetramethylcyclobutane-1,3-dithione (**11a**) was prepared from the dimethylketene dimer (2,2,4,4-tetramethylcyclobutane-1,3-dione) by thionation using tetraphosphorus decasulfide [37]. Adamantan-1-yl trifluoroacetate (CF_3_CO_2_Ad) was prepared following a known protocol [38]. 1-Bromoadamantane, 1-bromopentane (**12a**) and 1-bromododecane (**12b**) were commercial reagents used in the studied reactions with no additional purification.

**General procedure for the preparation of imidazole 3-oxides 7 (procedure A):** A solution of 5 mmol of **6** and 4.2 mmol of the corresponding α-hydroxyiminoketone **2** in 15 mL of glacial acetic acid was stirred magnetically overnight at rt. Then, 4.5 mL of conc. hydrochloric acid were added in small portions. Stirring was continued over 15 min, and after this time the acetic acid was evaporated. The semi-solid residue was triturated with a portion of diethyl ether and the crystalline material was separated by filtration. Next, the colorless crystals were dissolved in a portion of MeOH (10 mL) and while magnetically stirring, sodium hydrocarbonate was added in small portions until the evolution of carbon dioxide ceased. The mixture was stirred overnight, filtered and the filtrate was evaporated to dryness. The crude product was triturated again with diethyl ether and after few minutes filtered off. In some instances, products obtained thereby were dried over freshly prepared molecular sieves 4 Å to give hydrate-free samples.

**1-Adamantyloxy-4,5-dimethylimidazole 3-oxide** (**7a**): Yield: 204 mg (78%). Pale yellow crystals; mp 104–106 °C; ^1^H NMR δ 1.60, 1.69 (AB-system, *J*_H,H_ = 12.0 Hz, 6H, 3CH_2_(ad)), 1.85 (pseudo d, *J*_H,H_ = 2.6 Hz, 6H, 3CH_2_(ad)), 2.16, 2.19 (2s, 6H, 2CH_3_), 2.27 (brs, 3H, 3CH(ad)), 7.83 (s, 1H, HC(2)) ppm; ^13^C NMR δ 7.13, 8.47 (2CH_3_), 30.9 (3CH(ad)), 35.6, 40.7 (6CH_2_(ad)), 86.9 (C_q_(ad)-O), 120.9, 122.9, 123.6 (2C=, HC(2)) ppm; IR υ: 2909 (s), 2853 (m), 1523 (m), 1448 (m), 1355 (s), 1189 (m), 1082 (m), 1047 (vs), 963 (m), 881 (s), 738 (m), 574 (s) cm^−1^; anal. calcd for C_15_H_22_N_2_O_2_ (262.35): C, 68.67; H, 8.45; N, 10.68; found: C, 68.52; H, 8.56; N, 10.51.

**Deoxygenation of imidazole *N*-oxides 7a**–**d – general procedure:** A portion of freshly prepared Raney-Ni suspended in MeOH was added in excess to the stirred solution of 1 mmol of an imidazole *N*-oxide **7** dissolved in MeOH (ca. 1 mL). The solution was stirred magnetically for 1–2 h at rt. The progress of the deoxygenation was monitored by TLC. As soon as the starting material disappeared, the suspension was filtered and the filtrate was evaporated. The obtained material was triturated with petroleum ether and filtered off yielding analytically pure samples.

**1-Adamantyloxy-4,5-dimethyl-1*H*-imidazole** (**8a**): Yield: 96 mg (39%). Colorless crystals; mp 47–50 °C; ^1^H NMR δ 1.60, 1.67 (AB-system, *J*_H,H_ = 12.4 Hz, 6H, 3CH_2_(ad)), 1.85 (pseudo d, *J*_H,H_ = 2.5 Hz, 6H, 3CH_2_(ad)), 2.11, 2.13 (2s, 6H, 2CH_3_), 2.24 (brs, 3H, 3CH(ad)), 7.37 (s, 1H, HC(2)) ppm; ^13^C NMR δ 8.4, 13.3 (2CH_3_), 30.8 (3CH(ad)), 35.8, 41.0 (6CH_2_(ad)), 84.6 (C_q_(ad)-O), 121.4, 129.3, 131.8 (2C=, HC(2)) ppm; IR υ: 2905 (s), 2851 (m), 1679 (m), 1559 (m), 1440 (m), 1353 (m), 1295 (m), 1274 (m), 1095 (m), 1053 (vs), 896 (s), 799 (m), 769 (vs), 633 (m), 594 (m), 504 (s) cm^−1^; HRMS (ESI^+^): calcd. for [C_15_H_23_N_2_O]^+^, 247.1810; found, 247.1811.

**Sulfur-transfer reactions; Preparation of 3*H*-imidazole-2-thiones 10 – general procedure:** A solution of the corresponding imidazole *N*-oxide **7** (1 mmol) in 3 mL of CH_2_Cl_2_ was treated with 103 mg (0.6 mmol) of dithione **11a** and the obtained solution was stirred magnetically at rt for 1 h. The solvent was evaporated and the obtained residue was triturated with petroleum ether. The solid obtained thereby was filtered off and dried in the air.

**1-Adamantyloxy-4,5-dimethyl-1*H*-imidazole-2(3*H*)-thione** (**10a**): Yield: 103 mg (37%). Pale yellow crystals; mp 137–139 °C; ^1^H NMR δ 1.64 (pseudo t, *J*_H,H_ = 3.0 Hz, 6H, 3CH_2_(ad)), 2.06, 2.09 (2s, 6H, 2CH_3_), 2.13 (pseudo d, *J*_H,H_ = 3.1 Hz, 6H, 3CH_2_(ad)), 2.23 (brs, 3H, 3CH(ad)), 12.0 (brs, 1H, NH) ppm; ^13^C NMR δ 9.3, 9.4 (2CH_3_), 31.5 (3CH(ad)), 35.9, 42.0 (6CH_2_(ad)), 88.8 (C_q_(ad)-O), 117.4, 121.7 (2C=), 157.4 (C=S) ppm; IR υ: 3065 (m), 2907 (vs), 2853 (m), 1493 (vs), 1428 (m), 1399 (m), 1367 (m), 1248 (s), 1046 (vs), 889 (s), 900 (s), 773 (s), 743 (vs), 698 (vs), 577 (m) cm^−1^; HRMS (ESI^+^): calcd. for [C_15_H_23_N_2_OS]^+^, 279.1531; found, 279.1532.

**Preparation of non-symmetric 1,3-dialkoxyimidazolium bromides 13a**–**g – general procedure:** The corresponding imidazole *N*-oxide **7** (1 mmol) dissolved in 2–3 mL of chloroform was treated with alkylating reagent **12a** (227 mg, 1.50 mmol) or **12b** (262 mg, 1.05 mmol), which were added drop-wise at rt. The obtained reaction mixtures were stirred magnetically at rt and the progress of the reaction was monitored by TLC (SiO_2_, CH_2_Cl_2_). When the starting **7** was completely consumed, the solvent was evaporated and the residue was triturated with diethyl ether. In most cases, solid, colorless or beige colored products were formed. In the case of **13e**, a semi-solid product was formed; no crystallization was observed even after several days at rt. Similar properties were displayed by product **13d**, which did not crystallize at all and was analyzed as an oily material.

**1-Adamantyloxy-4,5-dimethyl-3-pentyloxy-1*H*-imidazolium bromide** (**13a**): Yield: 325 mg (79%). Beige crystals; mp 73 °C (dec.); ^1^H NMR δ 0.94 (t, *J*_H,H_= 7.1 Hz, 3H, CH_3_(p)), 1.38–1.40 (m, 2H, CH_2_(p)), 1.46–1.50 (m, 2H, CH_2_(p)), 1.69 (brs, 6H, 3CH_2_(ad)), 1.85–1.87 (m, 2H, CH_2_(p)), 2.02 (pseudo d, 6H, 3CH_2_(ad)), 2.27 (s, 3H, CH_3_-C=), 2.30 (s, 3H, CH_3_-C=), 2.35 (brs, 3H, 3CH(ad)), 4.79 (t, *J*_H,H_ = 6.5 Hz,2H, CH_2_-O), 11.46 (s, HC(2)) ppm; ^13^C NMR δ 7.3, 8.8, 13.9 (3CH_3_), 22.4, 27.5, 27.8 (3CH_2_(p)), 31.2 (3CH(ad)), 35.4, 40.7 (6CH_2_(ad)), 83.9 (CH_2_-O), 91.5 (C_q_(ad)-O), 120.0, 123.8 (2C=), 131.3 (HC(2)) ppm; IR υ: 2952 (s), 2911 (s), 1628 (m), 1373 (m), 1356 (m), 1039 (s), 958 (s), 877 (s), 593 (s) cm^−1^; HRMS (ESI^+^): calcd. for [C_20_H_33_N_2_O_2_]^+^, 333.2542; found, 333.2552.

**Preparation of symmetric imidazolium salt 15:** To a solution containing 145 mg of glyoxal (40% aqueous solution), adamantyloxyamine (**4**, 334 mg, 2 mmol), and paraformaldehyde (30 mg, 1 mmol) in 4.5 mL of glacial acetic acid, hydrobromic acid (2 mmol, 0.36 mL (32% solution in AcOH)) was added in one portion and the mixture obtained thereby was stirred magnetically overnight at rt. Next day, the solution was evaporated to dryness and the residue obtained as a thick oil was purified chromatographically on a short silica gel column using CH_2_Cl_2_/MeOH (97:3 mixture) as an eluent. The required product was isolated as a crystalline material. Additional crystallization from diisopropyl ether/CH_2_Cl_2_ mixture afforded analytically pure imidazolium salt **15**.

**1,3-Di(adamantyloxy)imidazolium bromide** (**15**): Yield: 190 mg (41%). Colorless crystals; mp ca. 160 °C (dec.); ^1^H NMR δ 1.59, 1.63 (AB-system, *J*_H,H_ = 12.5 Hz, 12H, 6CH_2_(ad)), 1.95 (pseudo d, *J*_H,H_ = 2.4 Hz 12H, 6CH_2_(ad)), 2.29 (brs, 6H, 6CH(ad)), 7.71 (pseudo d, *J*_H,H_ = 1.7 Hz, 2H, 2CH=), 11.03 (pseudo t, *J*_H,H_ = 1.9 Hz 1H, HC(2)) ppm; ^13^C NMR δ 31.0, 35.3, 40.2 (12CH_2_(ad)), 6CH(ad)), 90.8 (2C(ad)-O), 120.2 (2CH=), 133.7 (HC(2)) ppm; IR υ: 2903 (s), 2847 (m), 1546 (m), 1449 (m), 1358 (m), 1293 (m), 1045 (s), 1001 (s), 961 (s), 881 (s), 726 (s), 542 (vs), 449 (vs) cm^−1^; anal. calcd for C_23_H_33_BrN_2_O_2_∙H_2_O (467.44): C, 59.10; H, 7.55; N, 5.99; found: C, 59.08; H, 7.62; N, 5.79.

## Supporting Information

File 1 Experimental and analytical data and copies of NMR spectra.
