# Supplementary material for: Synthesis and selected transformations of 2-unsubstituted 1-(adamantyloxy)imidazole 3-oxides: straightforward access to non-symmetric 1,3-dialkoxyimidazolium salts
Source: Beilstein J Org Chem. 2019 Feb 19;15:497–505. doi: 10.3762/bjoc.15.43 (PMC6404403; doi:10.3762/bjoc.15.43)
Supplement: File 1 — Experimental and analytical data and copies of NMR spectra. [file Beilstein_J_Org_Chem-15-497-s001.pdf]

BEILSTEIN JOURNAL OF ORGANIC CHEMISTRY

Supporting Information

for

Synthesis and selected transformations of 2-unsubstituted 1-(adamantyloxy)imidazole 3-oxides: straightforward access to non-symmetric 1,3-dialkoxyimidazolium salts

Grzegorz Mlostoń, Małgorzata Celeda, Katarzyna Urbaniak, Marcin Jasiński, Vladyslav Bakhonsky, Peter R. Schreiner and Heinz Heimgartner

Beilstein J. Org. Chem. **2019,** *15,* 497–505. doi:10.3762/bjoc.15.43

Experimental and analytical data and copies of NMR spectra

License and Terms: This is a supporting information file under the terms of the Creative Commons Attribution License (http://creativecommons.org/licenses/by/4.0). Please note that the reuse, redistribution and reproduction in particular requires that the authors and source are credited.
The license is subject to the *Beilstein Journal of Organic Chemistry* terms and conditions: (https://www.beilstein-journals.org/bjoc)

Preparation of methylidene(adamantyl-1-oxy)amine (**6a**): To a solution of **4** (1.01 g, 6 mmol) in 20 mL of MeOH, paraformaldehyde (197 mg, 6.55 mmol) was added, and the mixture was heated to reflux for 1 h. Then, the solution was filtered and the clear filtrate was evaporated to dryness. Yield of **6a**: 961 mg (90%); mp 55–58 °C. An analytically pure sample was obtained by crystallization from diisopropyl ether after cooling in dry ice container. Colorless crystals; mp 61–62 °C; ^{1}H NMR δ 1.60, 1.69 (AB-system, $J_{H,H}$ = 12.0 Hz, 6H, 3CH_2(ad)), 1.84 (br. s, 6H, 3CH_2(ad)), 2.16 (br. s, 3H, 3CH(ad)), 6.39, 7.01 (AB-system, $J_{H,H}$ = 12 Hz, =CH_2) ppm; ^{13}C NMR δ 30.6 (3CH(ad)), 36.4 (3CH_2(ad)), 41.4 (3CH_2(ad)), 77.7 (C_q(ad)-O), 136.1 (N=CH_2)ppm; IR: υ 2905*vs*, 2851*s*, 1612*w* (C=N), 1483*m*, 1351*m*, 1108*m*, 1078*vs*, 948*br.vs*, 862*s*, 812*w* cm^{-1}; anal. calcd for $C_{11}H_{17}NO$ (179.26): C, 73.70; H, 9.56; N, 7.81; found: C, 73.86; H, 9.59; N, 7.90.

Imidazole *N*-oxides 7b–e:

1-Adamantyloxy-4,5-diphenylimidazole 3-oxide (**7b**)·H_2O: Yield: 380 mg (98%). Pale yellow crystals; mp 137–138 °C; ^{1}H NMR δ 1.46, 1.57 (AB-system, $J_{H,H}$ = 12.5 Hz, 6H, 3CH_2(ad)), 1.60 (pseudo d, $J_{H,H}$ = 2.5 Hz, 6H, 3CH_2(ad)), 2.13 (brs, 3H, 3CH(ad)), 7.32–7.38 (m, 8CH_{arom}), 7.55–7.57 (m, 2CH_{arom}), 8.10 (s, HC(2)) ppm; ^{13}C NMRδ 30.9 (3CH(ad)), 35.4, 40.6 (6CH_2(ad)), 88.4 (C_q(ad)-O), 124.3, 125.7, 126.4, 126.8, 127.2 (2C_{arom}, 2C=, HC(2)), 128.3, 128.5, 128.6, 129.1, 130.3, 130.4 (10CH_{arom}) ppm; IR: υ 2913*s*, 2853*m*, 1507*m*, 1448*m*, 1381*m*, 1358*m*, 1192*m*, 1045*s*, 883*s*, 857*s*, 762*vs*, 691*vs*, 599*m* cm^{-1}; anal. calcd for $C_{25}H_{26}N_2O_2\cdot H_2O$ (404.49): C, 74.23; H, 6.98; N, 6.92; found: C, 73.86; H, 7.03; N, 6.79.

1-Adamantyloxy-5-methyl-4-phenylimidazole 3-oxide (**7c**): Yield: 200 mg (62%). Pale yellow crystals; mp 121–122 °C; ^{1}H NMR δ 1.44, 1.55 (AB-system, $J_{H,H}$ = 12.5 Hz, 6H, 3CH_2(ad)), 1.57 (pseudo d, $J_{H,H}$ = 2.7 Hz, 6H, 3CH_2(ad)), 2.10 (brs, 3H, 3CH(ad)), 2.34 (s, 3H, CH_3), 7.46–7.48 (m, 2CH_{arom}), 7.49–7.51 (m, 3CH_{arom}), 7.97 (s, HC(2)) ppm;^{13}C NMR δ 7.9 (CH_3), 30.8 (3CH(ad)), 35.5, 40.6 (6CH_2(ad)), 87.9 (C_q(ad)-O), 123.5, 124.7, 125.1, 127.2 (C_{arom}, 2C=, HC(2)), 128.6, 128.8, 129.5

($5CH_{arom}$) ppm; IR: υ 2906*s*, 2851*m*, 1584*m*, 1444*m*, 1354*s*, 1232*m*, 1127*m*, 1053*s*, 900*s*, 857*m*, 768*vs*, 724*vs*, 602*s* cm^{-1}; anal. calcd for $C_{20}H_{24}N_2O_2$ (324.42): C, 74.04; H, 7.46; N, 8.64; found: C, 74.04; H, 7.34; N, 8.76.

1-Adamantyloxy-4-methyl-5-phenylimidazole 3-oxide (**7d**): Yield: 270 mg (83%). Pale yellow crystals; mp 130–132 °C; ^{1}H NMR δ 1.63, 1.72 (AB-system, $J_{H,H}$ = 12.5 Hz, 6H, $3CH_2$(ad)), 1.93 (pseudo d, $J_{H,H}$ = 2.7 Hz, 6H, $3CH_2$(ad)), 2.30 (s, 3H, CH_3), 2.32 (brs, 3H, 3CH(ad)), 7.37–7.40 (m, $1CH_{arom}$), 7.70–7.72 (m, $2CH_{arom}$), 7.97 (*s*, HC(2)) ppm; ^{13}C NMR δ 9.5 (CH_3), 30.9 (3CH(ad)), 35.6, 40.8 ($6CH_2$(ad)), 87.4 (C_q(ad)-O), 122.4, 123.8, 126.9, 127.1 (C_{arom}, 2C=, HC(2)), 128.3, 128.4, 129.5 ($5CH_{arom}$) ppm; IR: υ 2906*s*, 2851*m*, 1584*m*, 1496*m*, 1444*m*, 1354*s*, 1235*m*, 1127*m*, 1053*s*, 900*s*, 857*m*, 769*vs*, 698*vs*, 598*s* cm^{-1}; anal. calcd for $C_{20}H_{24}N_2O_2$ (324.42): C, 74.04; H, 7.46; N, 8.64; found: C, 74.01; H, 7.46; N, 8.75.

1-Adamantyloxy-4-[(4-bromophenyl)carbamoyl]-5-methylimidazole 3-oxide hydrochloride (**7e**)·$0.5H_2O$: Yield: 224 mg (50%). Pale yellow crystals; mp 197–199 °C; ^{1}H NMR δ 1.62, 1.71 (AB-system, $J_{H,H}$ = 12.4 Hz, 6H, $3CH_2$(ad)), 1.89 (pseudo d, $J_{H,H}$ = 2.7 Hz, 6H, $3CH_2$(ad)), 2.32 (brs, 3H, 3CH(ad)), 2.63 (s, 3H, CH_3), 7.44, 7.60 (AB-system, $J_{H,H}$ = 8.8 Hz, $4CH_{arom}$), 7.99 (s, HC(2)), 12.90 (s, NH) ppm; ^{13}C NMR δ 9.9 (CH_3), 31.0 (3CH(ad)), 35.5, 40.9 ($6CH_2$(ad)), 89.0 (C_q(ad)-O), 116.6, 119.0, 120.9, 123.8 (C_{arom}-Br, C_{arom}-N, 2C=), 122.0, 131.8 ($4CH_{arom}$), 157.3 (C=O) ppm; IR: υ 2909*m*, 2851*m*, 1675*m* (C=O), 1586*m*, 1552*s*, 1489*s*, 1314*m*, 1295*m*, 1049*s*, 821*s*, 803*vs*, 511*s* cm^{-1}; anal. calcd for $C_{21}H_{24}BrN_3O_3\cdot HCl\cdot 0.5H_2O$ (491.69): C, 51.28; H, 5.33; N, 8.54; found: C, 51.48; H, 5.48; N, 8.51.

Imidazoles 8b–d:

1-Adamantyloxy-4,5-diphenyl-1*H*-imidazole (**8b**): Yield: 244 mg (66%). Pale yellow crystals; mp 165–167 °C; ^{1}H NMR δ 1.47, 1.58 (AB-system, $J_{H,H}$ = 11.9 Hz, 6H, $3CH_2$(ad)), 1.59 (pseudo d, $J_{H,H}$ = 2.6 Hz, 6H, $3CH_2$(ad)), 2.11 (brs, 3H, 3CH(ad)), 7.21–7.22 (m, $1CH_{arom}$), 7.27–7.29 (m, $2CH_{arom}$), 7.37–7.42 (m, $3CH_{arom}$), 7.46–7.48 (m, $2CH_{arom}$), 7.55–7.57 (m, $2CH_{arom}$), 7.68 (*s*, HC(2)) ppm; ^{13}C NMR δ 30.8 (3CH(ad)), 35.7, 40.9 ($6CH_2$(ad)), 85.7 (C_q(ad)-O), 127.1, 128.2, 128.4, 130.6, 133.9

(10CH$_{arom}$), 127.0, 128.1, 129.7, 129.1, 134.8 (2C$_{arom}$, 2C=, HC(2)) ppm; IR: υ 2918*s*, 2853*m*, 1505*m*, 1457*m*, 1440*m*, 1353*m*, 1057*s*, 941*s*, 747*s*, 695*vs*, 587*s* cm^{-1}; HRMS (ESI$^+$): calcd. for $[C_{20}H_{27}N_2O]^+$: 371.2123; found 371.2126.

1-Adamantyloxy-5-methyl-4-phenyl-1*H*-imidazole (**8c**): Yield: 191 mg (62%). Pale yellow crystals; mp 105–107 °C; ^{1}H NMR δ 1.44, 1.54 (AB-system, $J_{H,H}$ = 12.0 Hz, 6H, 3CH$_2$(ad)), 1.58 (pseudo d, $J_{H,H}$ = 2.4 Hz, 6H, 3CH$_2$(ad)), 2.08 (brs, 3H, 3CH(ad)), 2.32 (s, 3H, CH$_3$), 7.32–7.33 (m, 1CH$_{arom}$), 7.41–7.44 (m, 2CH$_{arom}$), 7.47–7.49 (m, 2CH$_{arom}$), 7.51 (s, HC(2)) ppm; ^{13}C NMR δ 14.4 (CH$_3$), 30.8 (3CH(ad)), 35.7, 40.8 (6CH$_2$(ad)), 85.4 (C$_q$(ad)-O), 127.1, 128.2, 129.2 (5CH$_{arom}$), 126.4, 130.0, 131.2, 132.8 (C$_{arom}$, 2C=, HC(2)) ppm; IR: υ 2909*s*, 2849*m*, 1604*m*, 1492*m*, 1449*m*, 1351*s*, 1052*s*, 1025*s*, 883*s*, 754*vs*, 697*vs*, 596*s* cm^{-1}; anal. calcd for $C_{20}H_{24}N_2O$ (308.42): C, 77.89; H, 7.84; N, 9.08; found: C, 77.85; H, 7.92; N, 9.10.

1-Adamantyloxy-4-methyl-5-phenyl-1*H*-imidazole (**8d**): Yield: 120 mg (39%). Pale yellow crystals; mp 105–108 °C; ^{1}H NMR δ 1.63, 1.70 (AB-system, $J_{H,H}$ = 12.5 Hz, 6H, 3CH$_2$(ad)), 1.93 (pseudo d, $J_{H,H}$ = 2.6 Hz, 6H, 3CH$_2$(ad)), 2.28 (brs, 3H, 3CH(ad)), 2.43 (s, 3H, CH$_3$), 7.37–7.40 (m, 1CH$_{arom}$), 7.40–7.42 (m, 2CH$_{arom}$), 7.54 (s, HC(2)), 7.73–7.74 (m, 2CH$_{arom}$) ppm; ^{13}C NMR δ 10.3 (CH$_3$), 30.9 (3CH(ad)), 35.8, 41.0 (6CH$_2$(ad)), 85.3 (C$_q$(ad)-O), 126.1, 126.2, 128.4 (5CH$_{arom}$), 122.8, 132.8, 132.9, 135.3 (C$_{arom}$, 2C=, HC(2)) ppm; IR: υ 2914*s*, 2853*m*, 1602*m*, 1498*m*, 1442*m*, 1351*m*, 1299*m*, 1047*s*, 924*s*, 887*s*, 767*s*, 700*vs*, 578*m* cm^{-1}; HRMS (ESI$^+$): calcd. for $[C_{20}H_{25}N_2O]^+$: 309.1967; found 309.1974.

Isomerization of imidazole *N*-oxide 7b: A solution of 0.5 mmol (193 mg) of **7b** in 2 mL of $CHCl_3$ was stirred magnetically at rt and 0.25 mL of acetic anhydride was added drop-wise. The obtained solution was stirred overnight. Then, the solvent was evaporated and the solid residue was crystallized from a MeOH/CH_2Cl_2 mixture. The obtained crystals were filtered off and dried.

1-Adamantyloxy-4,5-diphenyl-3*H*-imidazol-2-one (9): Yield: 70 mg (36%). Colorless crystals; mp 119–120 °C; ^{1}H NMR δ 1.44, 1.46 (AB-system, $J_{H,H}$ = 12.0 Hz, 6H, 3CH$_2$(ad)), 1.67 (brs, 6H, 3CH$_2$(ad)), 2.03 (brs, 3H, 3CH(ad)), 7.15–7.45 (m,

10H, $2C_6H_5$), 11.0 (brs, 1H, NH) ppm; ^{13}C NMR δ 30.9 (3CH(ad)), 35.8 ($3CH_2$(ad)), 40.8 ($3CH_2$(ad)), 86.0 (C_q(ad)-O), 115.0, 121.0 (2C (imidazol)), 126.3, 127.1, 128.3, 128.4, 128.5, 131.0 ($10CH_{arom}$), 129.1, 129.8 ($2C_{arom}$), 153.0 (C=O) ppm; IR: υ 2907*m*, 2849*m*, 2771 (*br.m*, NH), 1705*vs* (C=O), 1444*m*, 1354*m*, 1298*w*, 1056*m*, 905*m*, 773*s*, 732*s*, 707*vs* cm^{-1}; HRMS (ESI$^+$): calcd. for $[C_{25}H_{27}N_2O_2]^+$: 387.2073; found 387.2073.

1*H*-Imidazole-2(3*H*)-thiones 10b–d:

1-Adamantyloxy-4,5-diphenyl-1*H*-imidazole-2(3*H*)-thione (**10b**): Yield: 220 mg (55%). Pale yellow crystals; mp 163–165 °C; ^{1}H NMR δ 1.45, 1.50 (AB-system, $J_{H,H}$ = 12.1 Hz, 6H, $3CH_2$(ad)), 1.71–1.80 (brs, 6H, $3CH_2$(ad)), 2.01 (brs, 3H, 3CH(ad)), 7.27–7.31 (m, $3CH_{arom}$), 7.33–7.36 (m, $2CH_{arom}$), 7.39–7.44 (m, $5CH_{arom}$), 12.12 (brs, 1H, NH) ppm; ^{13}C NMR δ 31.4 (3CH(ad)), 35.7, 41.6 ($6CH_2$(ad)), 90.3 (C_q(ad)-O), 127.3, 128.2, 128.6, 128.7, 129.2, 131.0 ($10CH_{arom}$), 122.3, 126.7, 128.0, 129.1 ($2C_{arom}$, 2C=), 159.7 (C=S) ppm; IR: υ 3062*m*, 2907*s*, 2849*m*, 1477*s*, 1448*m*, 1388*m*, 1353*m*, 1295*s*, 1049*m,* 877*s*, 900*s*, 733*s*, 695*vs*, 564*m* cm^{-1}; anal. calcd for $C_{25}H_{26}N_2OS$ (402.55): C, 74.59; H, 6.51; N, 6.96; S, 7.97; found: C, 74.75; H, 6.79; N, 6.87; S, 7.78.

1-Adamantyloxy-5-methyl-4-phenyl-1*H*-imidazole-2(3*H*)-thione (**10c**): Yield: 300 mg (88%). Pale yellow crystals; mp 153–154 °C; ^{1}H NMR δ 1.45, 1.50 (AB-system, $J_{H,H}$ = 12.2 Hz, 6H, $3CH_2$(ad)), 1.78 (pseudo d, $J_{H,H}$ = 2.2 Hz, 6H, $3CH_2$(ad)), 2.05 (brs, 3H, 3CH(ad)), 2.22 (s, 3H, CH_3), 7.35–7.39 (m, $1CH_{arom}$), 7.42–7.45 (m, $4CH_{arom}$), 12.49 (brs, 1H, NH) ppm; ^{13}C NMR δ 10.1 (CH_3), 31.3 (3CH(ad)), 35.7, 41.6 ($6CH_2$(ad)), 90.0 (C_q(ad)-O), 119.0, 126.7, 128.2 (C_{arom}, 2C=), 128.3, 128.4, 129.7 ($5CH_{arom}$), 157.7 (C=S) ppm; IR: υ 3062*m*, 2905*s*, 2851*m*, 1502*s*, 1478*m*, 1448*m*, 1377*m*, 1297*m*, 1244*m*, 1042*s,* 977*m*, 933*s*, 756*vs*, 698*vs*, 575*m* cm^{-1}; HRMS (ESI$^+$): calcd. for $[C_{20}H_{25}N_2OS]^+$: 341.1688; found 341.1691.

1-Adamantyloxy-4-methyl-5-phenyl-1*H*-imidazole-2(3*H*)-thione (**10d**): Yield: 99 mg (29%). Pale yellow crystals; mp 165–166 °C; ^{1}H NMR δ 1.66 (pseudo t, $J_{H,H}$ = 2.9 Hz, 6H, $3CH_2$(ad)), 2.21 (pseudo d, $J_{H,H}$ = 3.1 Hz, 6H, $3CH_2$(ad)), 2.26 (brs, 3H, 3CH(ad)), 2.37 (s, 3H, CH_3), 7.31–7.34 (m, $1CH_{arom}$), 7.43–7.46 (m, $2CH_{arom}$), 7.50–

7.52 (m, 2CH_{arom}), 11.92 (brs, 1H, NH) ppm; ^{13}C NMR δ 10.8 (CH_3), 31.5 (3CH(ad)), 35.8, 42.0 (6CH_2(ad)), 89.6 (C_q(ad)-O), 121.9, 122.9, 128.6 (C_{arom}, 2C=), 126.7, 127.9, 129.0 (5CH_{arom}), 159.2 (C=S) ppm; IR: υ 3049*m*, 2912*s*, 2848*m*, 1569*m*, 1489*m*, 1433*m*, 1357*m*, 1297*m*, 1244*m*, 1041*s,* 881*s*, 933*s*, 756*vs*, 698*vs*, 525*m* cm^{-1}; anal. calcd for $C_{20}H_{24}N_2OS$ (340.48): C, 70.55; H, 7.10; N, 8.23; S, 9.42; found: C, 70.56; H, 7.08; N, 8.23; S, 9.50.

3-Alkoxyimidazolium bromides 13b–g:

1-Adamantyloxy-3-dodecyloxy-4,5-dimethyl-1*H*-imidazolium bromide (**13b**): Yield: 415 mg (81%). Pale yellow crystals; mp 116 °C (decomp.); ^1H NMR δ 0.94 (*t*, $J_{H,H}$= 6.9 Hz, 3H, CH_3(d)), 1.23–1.33 (m, 14H, 7CH_2(d)), 1.46–1.52 (m, 2H, CH_2(d)), 1.81–1.86 (m, 4H, 2CH_2(d)), 1.69 (brs, 6H, 3CH_2(ad)), 2.01 (pseudo d, $J_{H,H}$ = 2.6 Hz, 6H, 3CH_2(ad)), 2.28 (s, 3H, CH_3-C=), 2.30 (s, 3H, CH_3-C=), 2.34 (brs, 3H, 3CH(ad)), 4.80 (t, $J_{H,H}$ = 6.9 Hz, 2H, CH_2-O), 11.50 (s, HC(2)) ppm; ^{13}C NMR δ 7.3, 8.3, 14.1 (3CH_3), 22.7, 25.6, 27.8, 29.3, 29.4, 29.5, 29.6, 29.7, 29.8, 31.9 (10CH_2(d)), 31.3 (3CH(ad)), 35.3, 40.8 (6CH_2(ad)), 83.9 (CH_2-O), 91.4 (C_q(ad)-O), 122.0, 123.7 (2C=), 131.4 (HC(2)) ppm; IR: υ 2914*s*, 2849*s*, 1638*m*, 1468*m*, 1399*m*, 1295*m*, 1049*s*, 1003m, 892*s*, 589*s* cm^{-1}; HRMS (ESI^+): calcd. for $[C_{27}H_{47}N_2O_2]^+$: 431.3638; found 431.3651.

1-Adamantyloxy-3-pentyloxy-4,5-diphenyl-1*H*-imidazolium bromide (**13c**): Yield: 425 mg (79%). Colorless crystals; mp 158 °C (decomp.); ^1H NMR δ 0.79 (t, $J_{H,H}$ = 6.9 Hz, 3H, CH_3), 1.14–1.20 (m, 4H, 2CH_2(p)), 1.50–1.59 (m, 6H, 3CH_2(ad)), 1.65–1.68 (m, 2H, CH_2(p)), 1.74 (brd, 6H, 3CH_2(ad)), 2.19 (brs, 3H, 3CH(ad)), 4.61 (t, $J_{H,H}$ = 7.0 Hz, 2H, CH_2-O), 7.38–7.53 (m, 10CH_{arom}), 12.12 (s, HC(2)) ppm; ^{13}C NMR δ 13.7 (CH_3), 22.1, 27.3, 27.4 (3CH_2(p)), 31.2 (3CH(ad)), 35.3, 40.5 (6CH_2(ad)), 84.3 (CH_2-O), 92.3 (C_q(ad)-O), 122.7, 123.9, 125.8, 127.9 (2C_{arom}, 2C=), 129.1, 129.2, 130.2, 130.4, 130.6, 130.7 (10CH_{arom}), 132.8 (HC(2)) ppm; IR: υ 3035*m*, 2914*m*, 2853*m*, 1647*m*, 1448*m*, 1358*m*, 1295*m*, 1053*s*, 967*s*, 793*s*, 589*s* cm^{-1}; anal. calcd for $C_{30}H_{37}BrN_2O_2$ (536.20): C, 67.03; H, 6.94; N, 5.21; found: C, 67.20; H, 7.04; N, 5.25.

1-Adamantyl-4,5-dimethyl-3-pentyloxy-1*H*-imidazolium bromide (**13d**): Yield: 310 mg (78%). Semi-solid beige crystals; ^1H NMR δ 0.90 (t, $J_{H,H}$= 7.3 Hz, 3H, CH_3(p)), 1.31–1.42 (m, 2H, CH_2(p)), 1.43–1.49 (m, 2H, CH_2(p)), 1.73 (pseudo t, 6H, $3CH_2$(ad)), 1.80–1.87 (m, 2H, CH_2(p)), 2.23 (s, 3H, CH_3-C=), 2.29 (brs, 3H, 3CH(ad)), 2.33 (s, 6H, CH_2(ad)), 2.46 (s, 3H, CH_3-C=), 4.69 (t, $J_{H,H}$ = 6.7 Hz, 2H, CH_2-O), 10.24 (brs, 1H, HC(2)) ppm; ^{13}C NMR δ 7.1 (CH_3), 12.6, 13.8 (2CH(ad)), 22.4, 27.5, 27.6 ($3CH_2$(p)), 29.6 (CH(ad)), 35.3 ($3CH_2$(ad)), 41.6 ($3CH_2$(ad)), 64.0 (C(ad)-N), 83.4 (CH_2-O), 123.6, 125.7 (2C=), 131.4 (HC(2)) ppm; IR: υ 2913*s*, 2855*s*, 1451*m*, 1304*m*, 983*s*, 723*vs*, 625*m* cm^{-1}; HRMS (ESI^+): calcd. for $[C_{20}H_{33}N_2O]^+$: 317.2593; found 317.2605.

1-Adamantyl-3-dodecyloxy-4,5-dimethyl-1*H*-imidazolium bromide (**13e**): Yield: 370 mg (75%). Beige crystals; mp 100–104 °C; ^1H NMR δ 0.88 (t, $J_{H,H}$= 6.8 Hz, 3H, CH_3(d)), 1.26–1.34 (m, 16H, $8CH_2$(d)), 1.46–1.49 (m, 2H, CH_2(d)), 1.75–1.79 (m, 6H, $3CH_2$(ad)), 1.81–1.86 (m, 2H, CH_2(d)), 2.25 (s, 3H, CH_3-C=), 2.33 (brs, 3H, 3CH(ad)), 2.38 (s, 6H, CH_2(ad)), 2.48 (s, 3H, CH_3-C=), 4.78 (t, $J_{H,H}$ = 6.5 Hz, 2H, CH_2-O), 10.24 (brs, 1H, HC(2)) ppm; ^{13}C NMR δ 7.0, 12.5, 14.2 ($3CH_3$), 25.6, 27.6, 27.9, 29.0, 29.3, 29.4, 29.5, 29.7, 29.8 ($10CH_2$(d)), 31.9 (3CH(ad)), 35.3, 41.6 ($6CH_2$(ad)), 64.1 (C_q(ad)-N), 83.5 (CH_2-O), 123.3, 125.6 (2C=), 131.7 (HC(2)) ppm; IR: υ 2918*s*, 2849*s*, 1531*m*, 1453*m*, 1306*m*, 1226*m*, 1187*m*, 1076*m*, 944*m*, 635*m* cm^{-1}; anal. calcd for $C_{27}H_{47}BrN_2O$ (495.58): C,65.44; H, 9.56; N, 5.65; found: C, 65.65; H, 9.62; N, 5.86.

1-Adamantyl-3-pentyloxy-4,5-diphenyl-1*H*-imidazolium bromide (**13f**): Yield: 400 mg (77%). Beige crystals; mp 181 °C (dec.); ^1H NMR δ 0.78 (t, $J_{H,H}$ = 6.8 Hz, 3H, CH_3), 1.13–1.19 (m, 4H, $2CH_2$(p)), 1.59–1.65 (m, 6H, $3CH_2$(ad)), 1.78 (brs, 2H, CH_2(p)), 2.18 (brs, 3H, 3CH(ad)), 2.33 (brs, 6H, $3CH_2$(ad)), 4.63 (t, $J_{H,H}$ = 6.8 Hz, 2H, CH_2-O), 7.29–7.51 (m, $10CH_{arom}$), 10.68 (s, HC(2)) ppm; ^{13}C NMR δ 13.8 (CH_3), 22.1, 27.4 ($3CH_2$(p)), 29.8 (3CH(ad)), 35.3, 42.6 ($6CH_2$(ad)), 66.6 (C_q(ad)-N), 83.5 (CH_2-O), 128.6, 128.7, 129.8, 130.1, 130.7, 132.5 ($10CH_{arom}$), 123.1, 127.5 130.1, 130.7 ($2C_{arom}$, 2C=), 132.7 (HC(2)) ppm; IR: υ 2938*s*, 2854*s*, 1525*m*, 1499*m*, 1445*m*, 1365*m*, 1325*m*, 1246*m*, 1167*m*, 1147*m*, 987*m*, 763*s*, 696*s* cm^{-1}; anal. calcd for $C_{30}H_{37}BrN_2O$ (520.21): C, 69.09; H, 7.15; N, 5.37; found: C, 69.11; H, 7.05; N, 5.30.

1-Adamantyl-3-dodecyloxy-4,5-diphenyl-1*H*-imidazolium bromide (**13g**): Yield: 460 mg (74%). Beige colored powder; mp 73–75 °C; ^{1}H NMR δ 0.83 (t, $J_{H,H}$= 6.6 Hz, 3H, CH_3(d)), 1.03−1.23 (m, 20H, 10CH_2(d)), 1.53–1.59 (m, 6H, 3CH_2(ad)), 2.12 (brs, 3H, 3CH(ad)), 2.28 (pseudo d, $J_{H,H}$ = 1.9 Hz, 6H, 3CH_2(d)), 4.55 (t, $J_{H,H}$ = 6.5 Hz, CH_2-O), 7.21–7.42 (m, 10CH_{arom}), 10.6 (brs, HC(2)) ppm; ^{13}C NMR δ 14.0 (CH_3), 22.6, 25.3, 27.7, 29.1, 29.3, 29.4, 29.8, 31.9, 32.8, 34.0 (10CH_2(d)), 30.1 (3CH(ad)), 35.0, 42.5 (6CH_2(ad)), 66.5 (C(ad)-N), 83.5 (CH_2-N), 123.0, 127.3, 130.1, 130.5 (2C_{arom}, 2C=), 128.6, 128.7, 129.8, 130.7, 132.4 (10CH_{arom}), 132.5 (HC(2)) ppm; IR: υ 2918*s*, 2853*s*, 1521*m*, 1457*m*, 1306*m*, 1228*m*, 1167*m*, 1021*m*, 940*m*, 783*s*, 699*vs* cm^{-1}; anal. calcd for $C_{37}H_{51}BrN_2O$ (619.72): C, 71.71; H, 8.29; N, 4.52; found: C, 71.45; H, 8.31; N, 4.21.

Attempted O-adamantylation of imidazole *N*-oxide 7a with adamantan-1-yl trifluoroacetate: In a 5 mL round bottom flask, imidazole *N*-oxide **7a** (131.0 mg, 0.5 mmol) was treated with a 5-fold excess of freshly prepared adamantan-1-yl trifluoroacetate (617 mg) and the obtained slurry was stored at rt overnight. The next day, the semi-solid material was triturated with 5 mL of pentane and the crystalline product **14** formed was filtered off. The filtrate was cooled in a refrigerator, and next day the separated white crystals of adamantan-1-ol (45 mg, 52%) were filtered off and identified by comparison with an original sample.

1-Adamantyloxy-3-hydroxy-4,5-dimethyl-1*H*-imidazolium trifluoroacetate (**14**): Yield: 115 mg (62%). Colorless crystals; mp 165 °C (dec.); ^{1}H NMR δ 1.64, 1.72 (AB-system, $J_{H,H}$ = 12.0 Hz, 6H, 3CH_2(ad)), 1.90 (pseudo d, $J_{H,H}$ = 2.6 Hz, 6H, 3CH_2(ad)), 2.24, 2.27 (2s, 6H, 2CH_3), 2.27 (brs, 3H, 3CH(ad)), 8.70 (s, 1H, HC(2)) ppm; ^{13}C NMR δ 7.2, 8.3 (2CH_3), 31.1 (3CH(ad)), 35.4, 40.6 (6CH_2(ad)), 89.8 (C_q(ad)-O), 116.0 (q, $^{1}J_{C,F}$ = 289.4 Hz, CF_3), 122.6, 123.7, 126.3 (2C=, HC(2)), 161.6 (q, $^{2}J_{C,F}$ = 36.1 Hz, C=O) ppm;IR: υ 2917*s*, 2853*w*, 1741*m*, br., 1364*m*, 1166*vs*, 1116*vs*, 1086*s*, 1040*s*, 875*m*, 616*m*, 715*vs*, 551*m*, 464*m* cm^{-1}; anal. calcd for $C_{17}H_{23}F_3N_2O_4$ (376.37): C, 54.25; H, 6.16; N, 7.44; found: C, 54.14; H, 6.30; N, 7.40.

Trapping of the in situ generated 1,3-di(adamantyloxy)imidazole-2-ylidene 16 with elemental sulfur: A solution of imidazolium salt **15** (449 mg, 1 mmol) in 10 mL

of dry pyridine was treated with 100 mg (1 mmol) triethylamine and elemental sulfur 32 mg (1 mmol). The solution was stirred magnetically at rt for 1 h and the solvents were evaporated in vacuo. The residue obtained thereafter was separated on preparative plates (SiO_2) using dichloromethane as an eluent. The isolated product was purified by crystallization from petroleum ether with a small amount of dichloromethane.

1,3−Di(adamantyloxy)-1*H*-imidazole-2(3*H*)-thione (**17**): Yield: 330 mg (83%). Pale yellow crystals; mp = 171−173 °C; ^{1}H NMR δ 1.59−1.69 (*m*, 3CH_2(ad)), 2.06 (*pseudo d*, $J_{H,H}$ = 2.6 Hz, 6H, 3CH_2(ad)), 2.26 (*brs*, 3H, 3CH(ad)), 6.69 (*brs*, 2H, H(4) and H(5)); ^{13}C NMR δ 31.0 (3CH(ad)), 35.9, 41.4 (6CH_2(ad)), 87.9 (C_q(ad)-O), 114.6 (C(4), C(5)), 161.9 (C=S); IR: υ 3058*m*, 2903*s*, 2849*m*, 1448*m*, 1388*s*, 1351*s*, 1293*m*, 1244*m*, 1187*m*, 1049*s*, 998*s*, 810*m*, 676*vs*, 570*m*; anal. calcd for $C_{23}H_{32}N_2O_2S$ (400.58): C, 68.96; H, 8.05; N, 6.99; S, 8.00; found: C, 68.77; H, 8.18; N,7.06; S, 7.94.

Collected ^{1}H and ^{13}C NMR spectra for compounds 6, 7, 8, 10, 13, 14, and 15

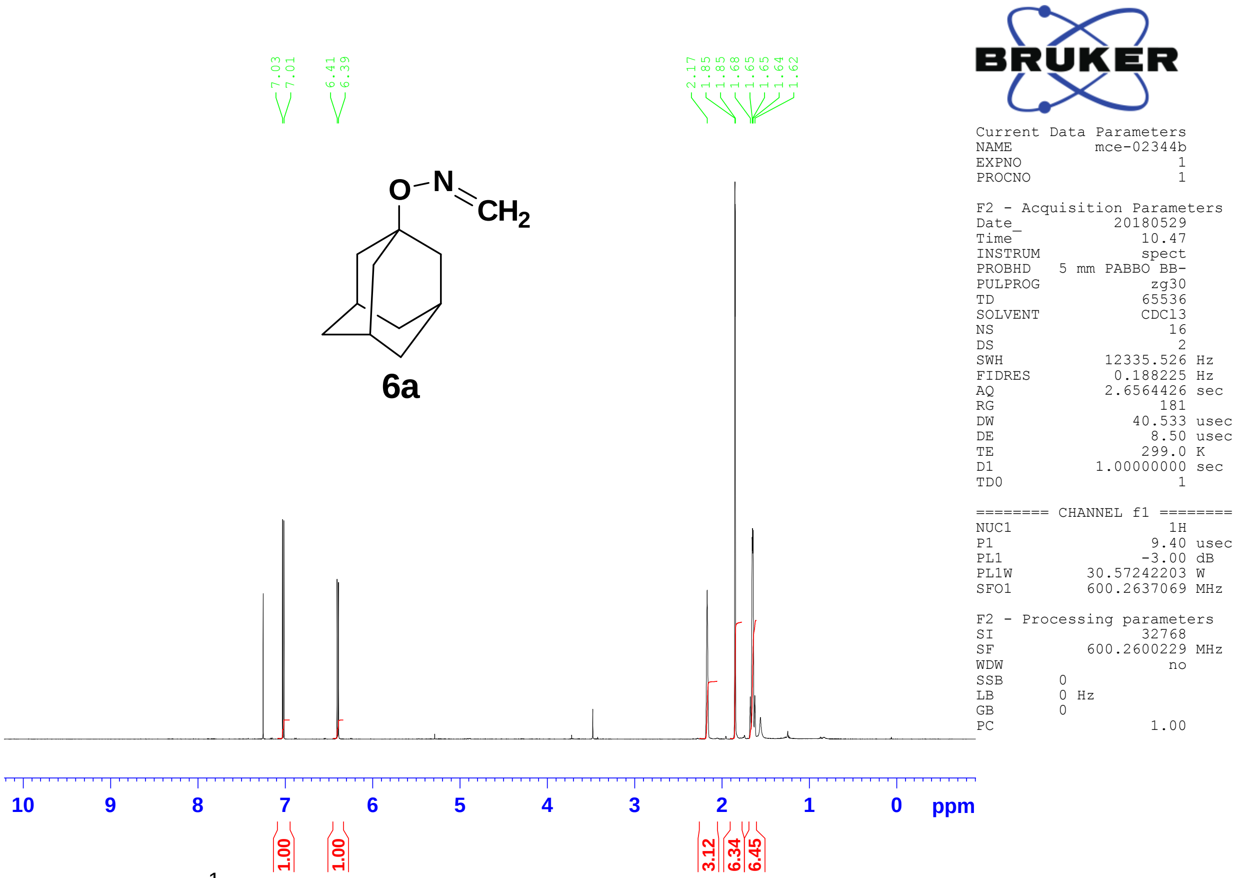

Fig. S1a. The ^{1}H NMR spectrum for compound **6a**.

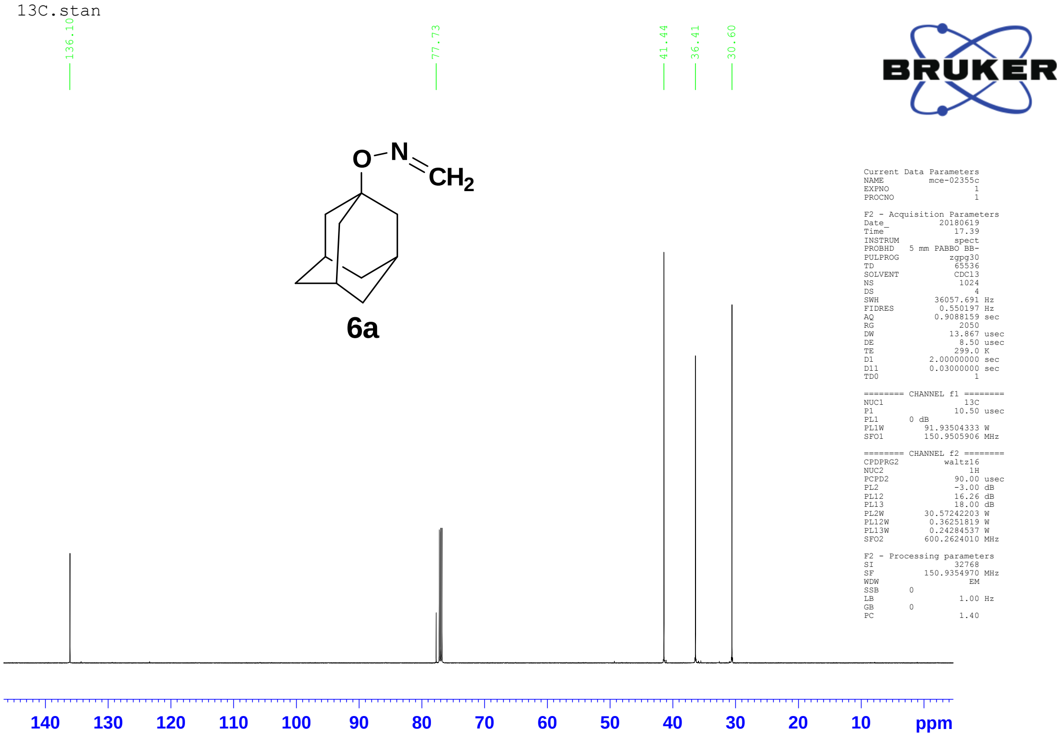

Fig. S1b. The ^{13}C NMR spectrum for compound **6a**.

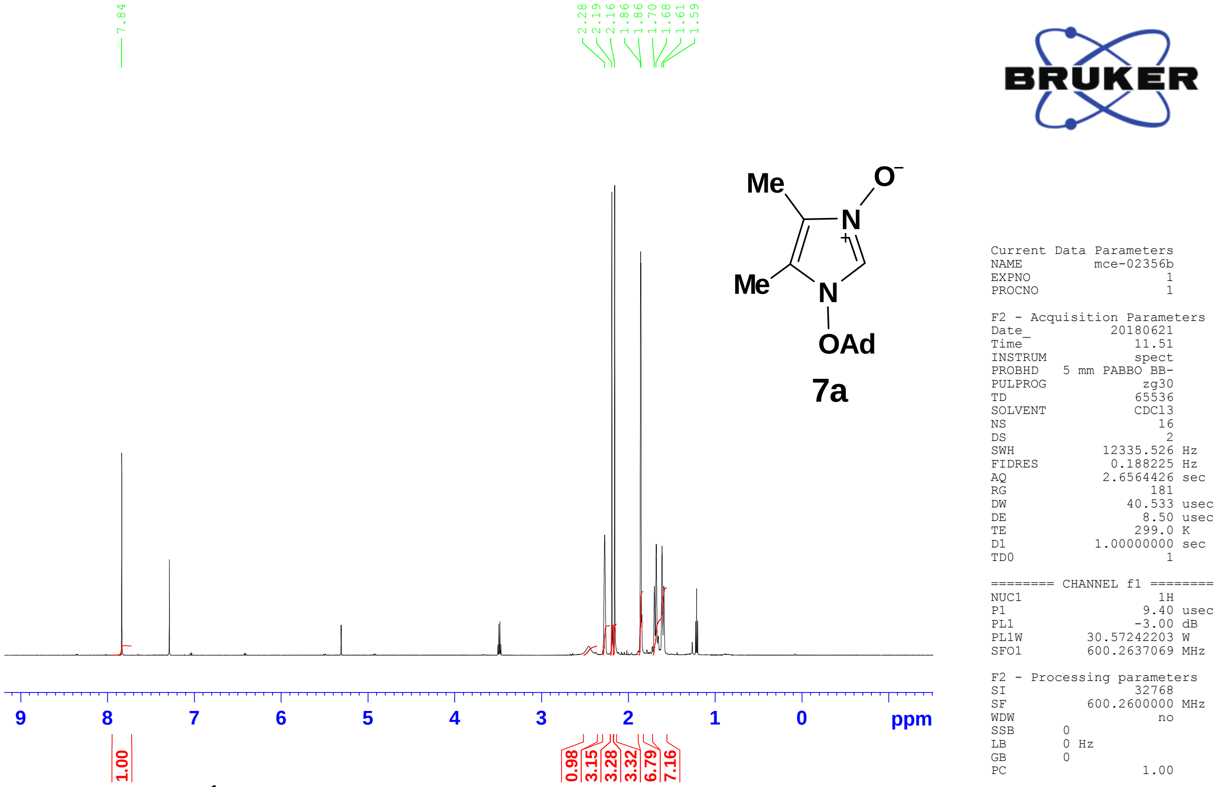

Fig. S2a. The ^{1}H NMR spectrum for compound **7a.**

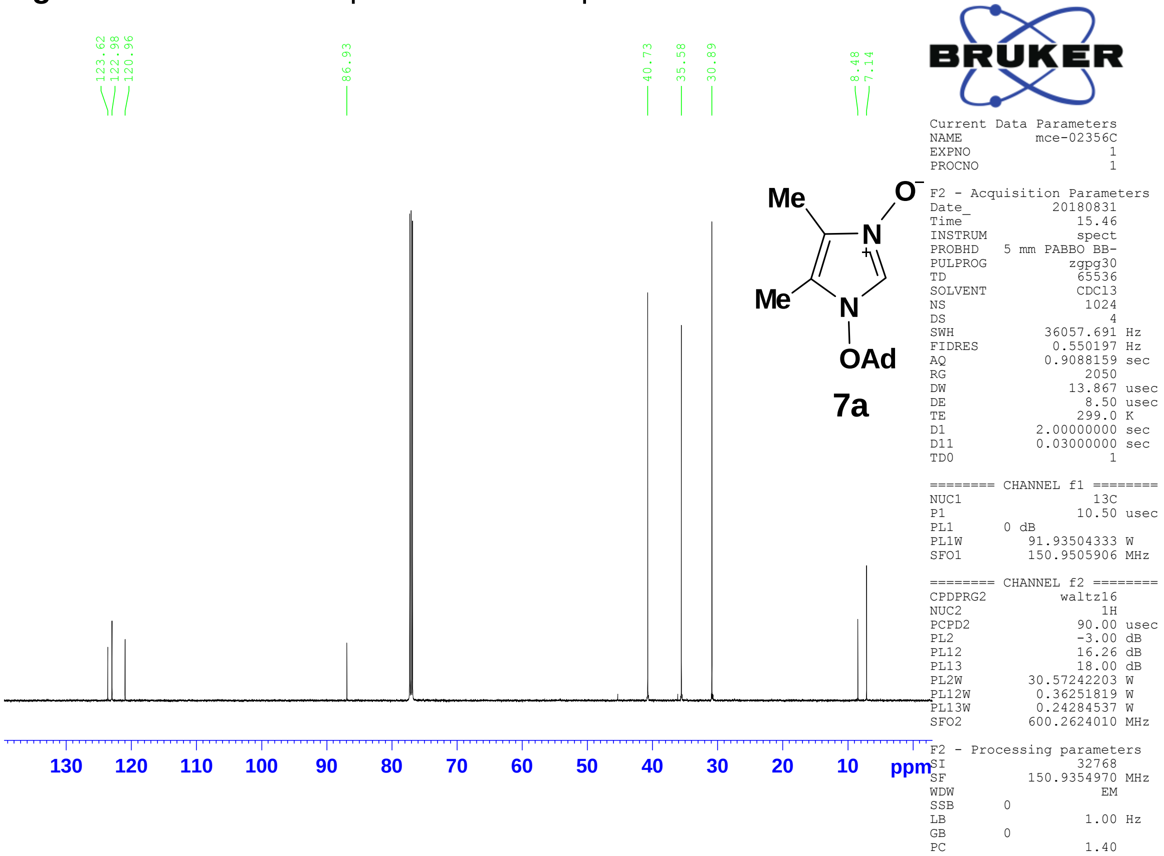

Fig. S2b. The ^{13}C NMR spectrum for compound **7a.**

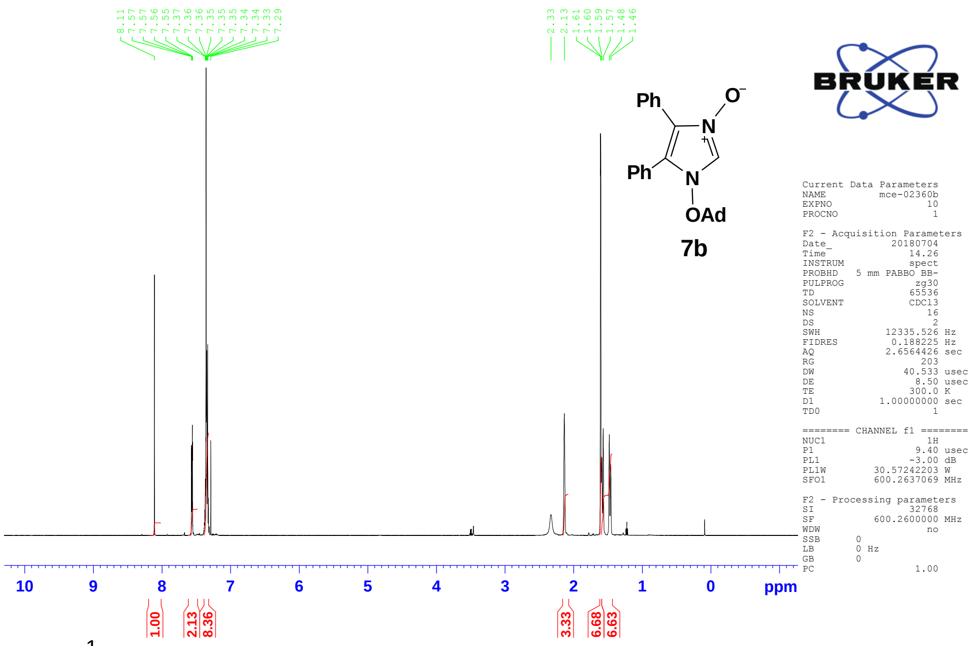

Fig. S3a. The ^{1}H NMR spectrum for compound **7b**.

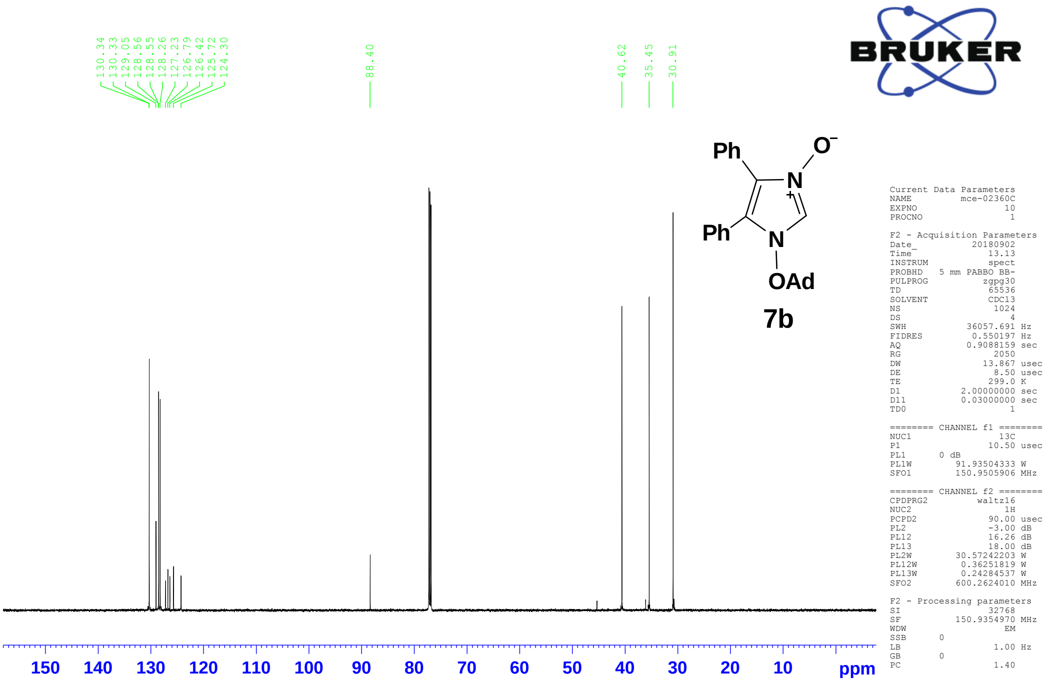

Fig. S3b. The ^{13}C NMR spectrum for compound **7b**.

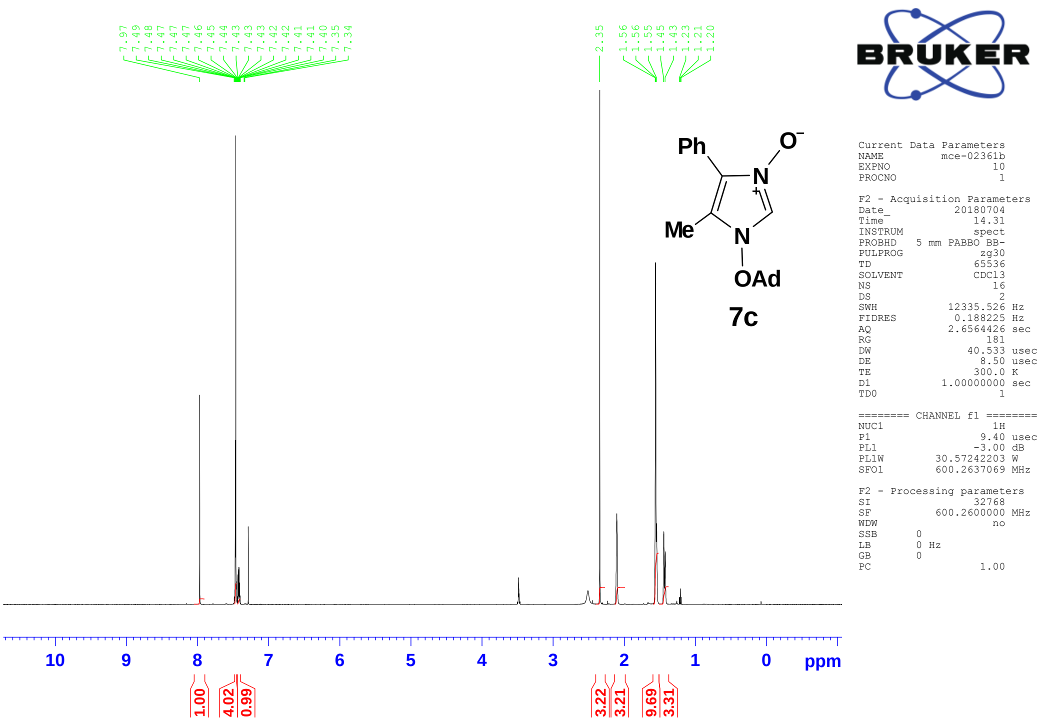

Fig. S4a. The ^{1}H NMR spectrum for compound **7c**.

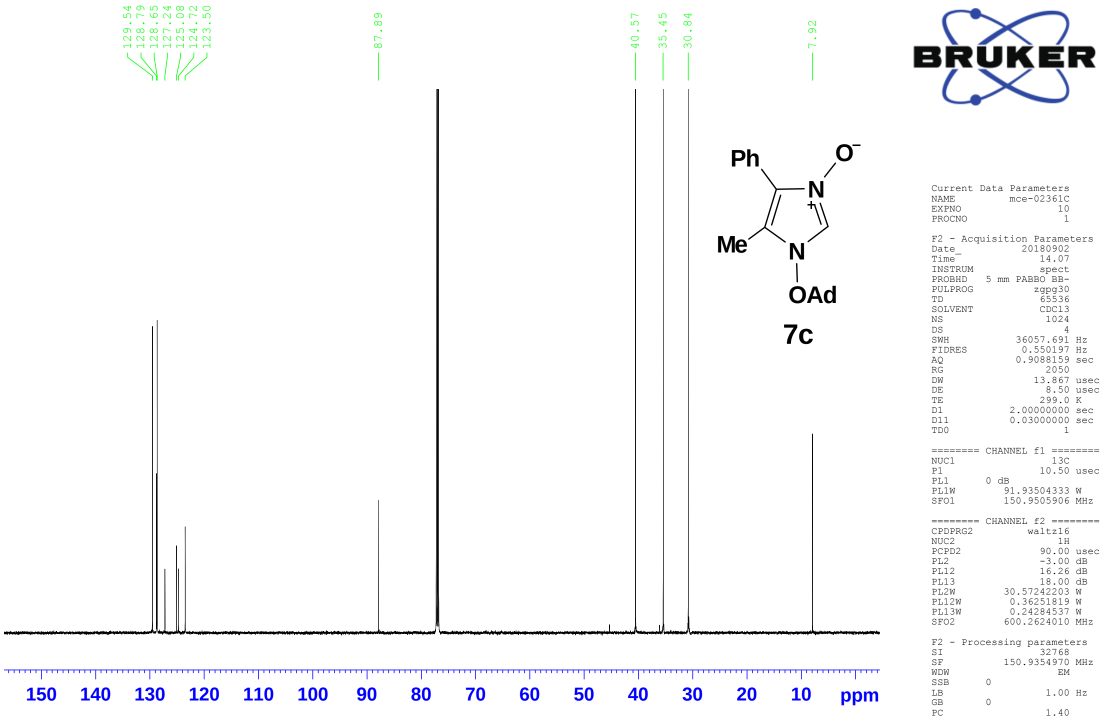

Fig. S4b. The ^{13}C NMR spectrum for compound **7c**.

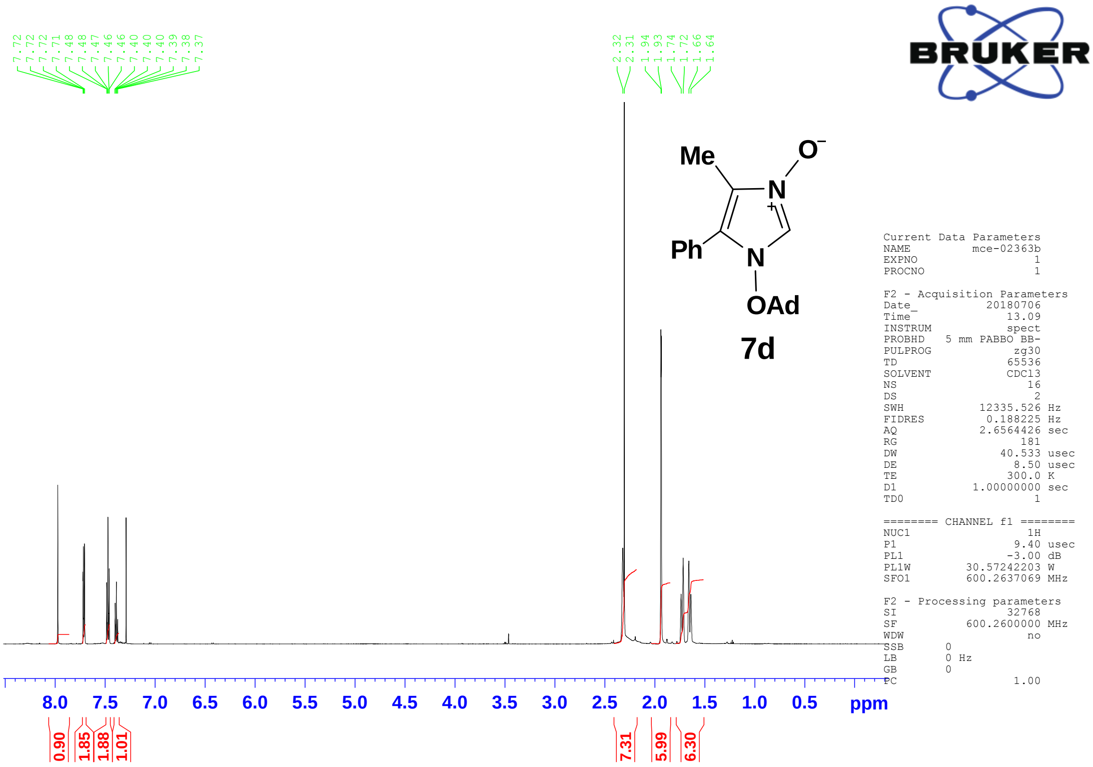

Fig. S5a. The ^{1}H NMR spectrum for compound **7d**.

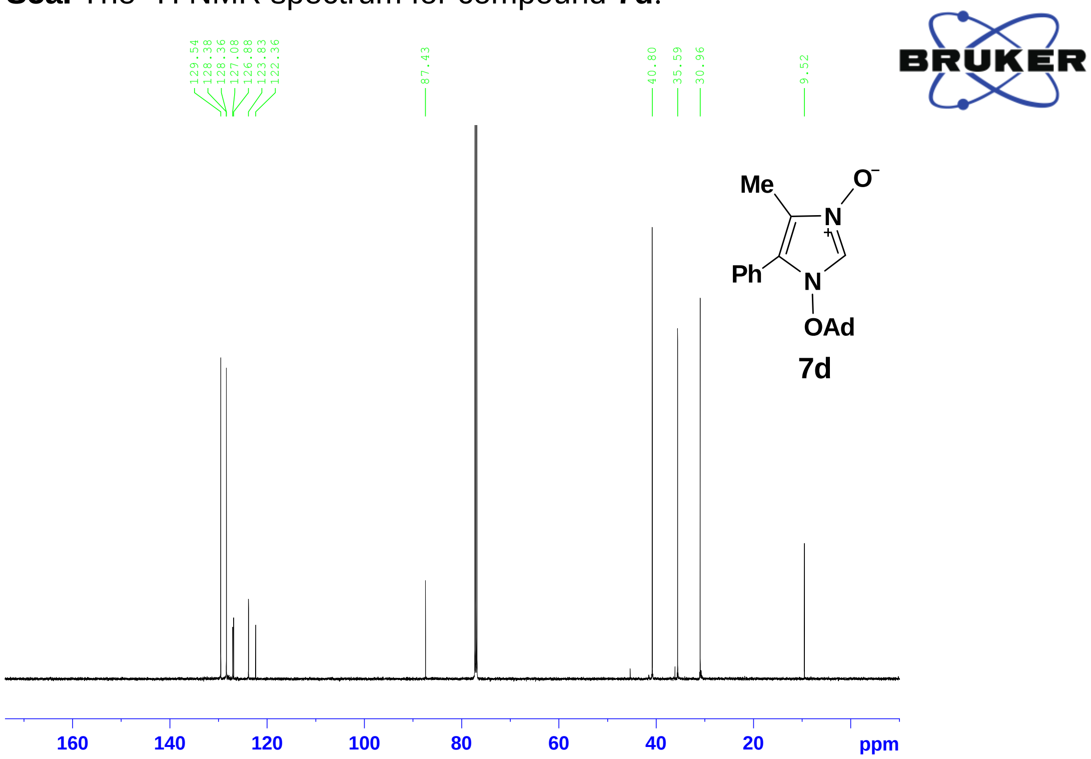

Fig. S5b. The ^{13}C NMR spectrum for compound **7d**.

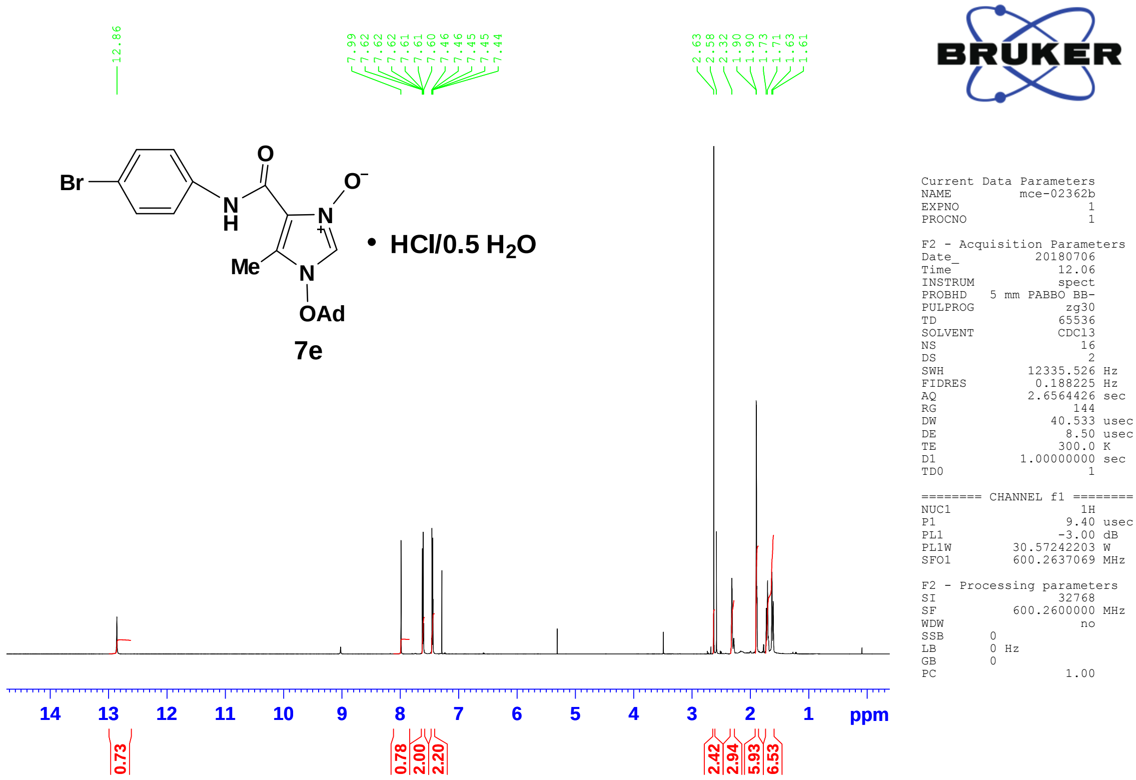

Fig. S6a. The ^{1}H NMR spectrum for compound **7e**.

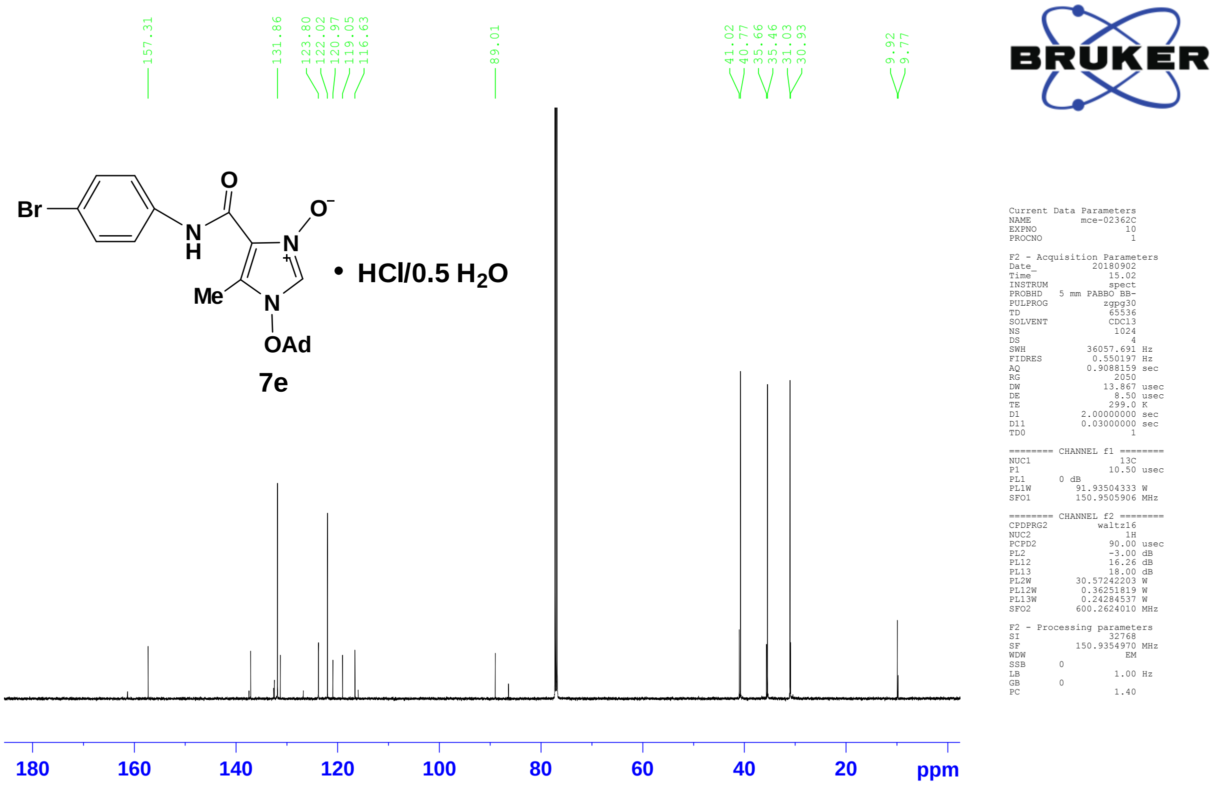

Fig. S6b. The ^{13}C NMR spectrum for compound **7e**.

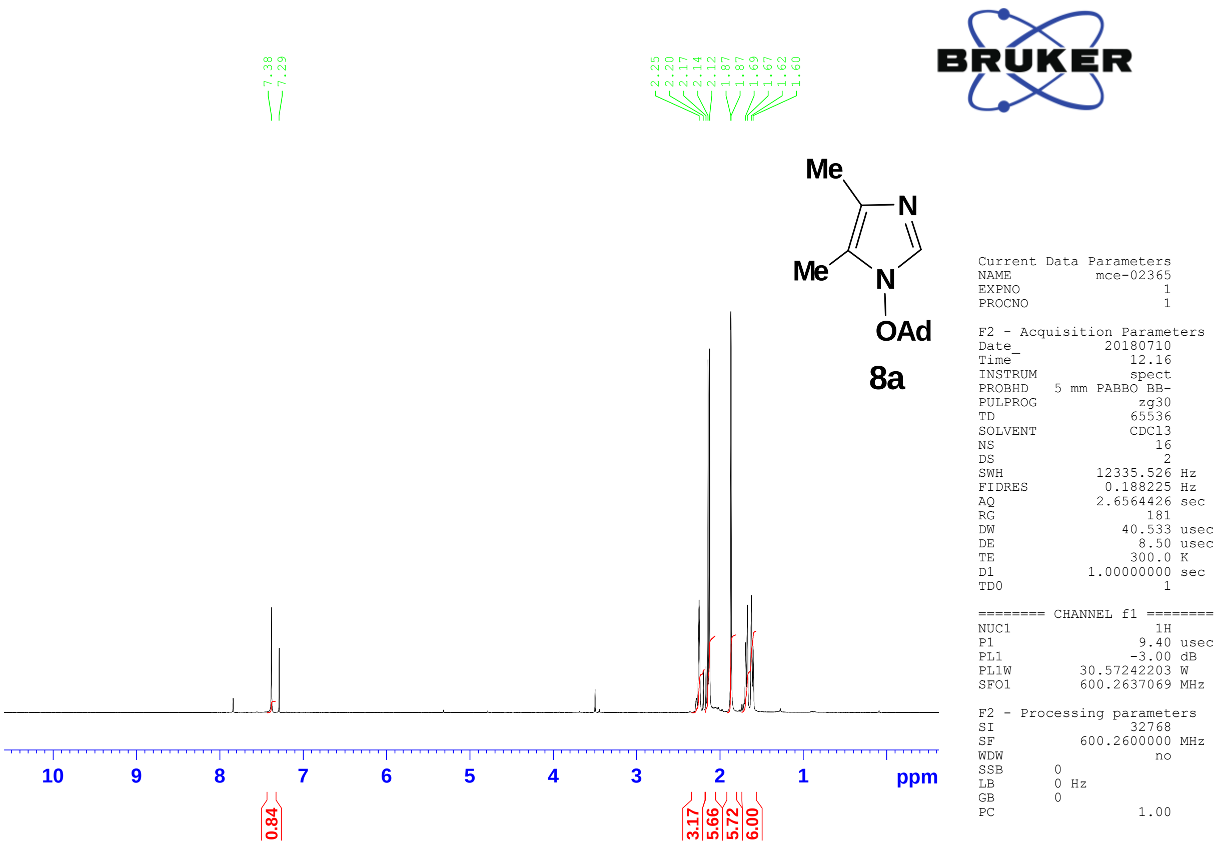

Fig. S7a. The ^{1}H NMR spectrum for compound **8a**.

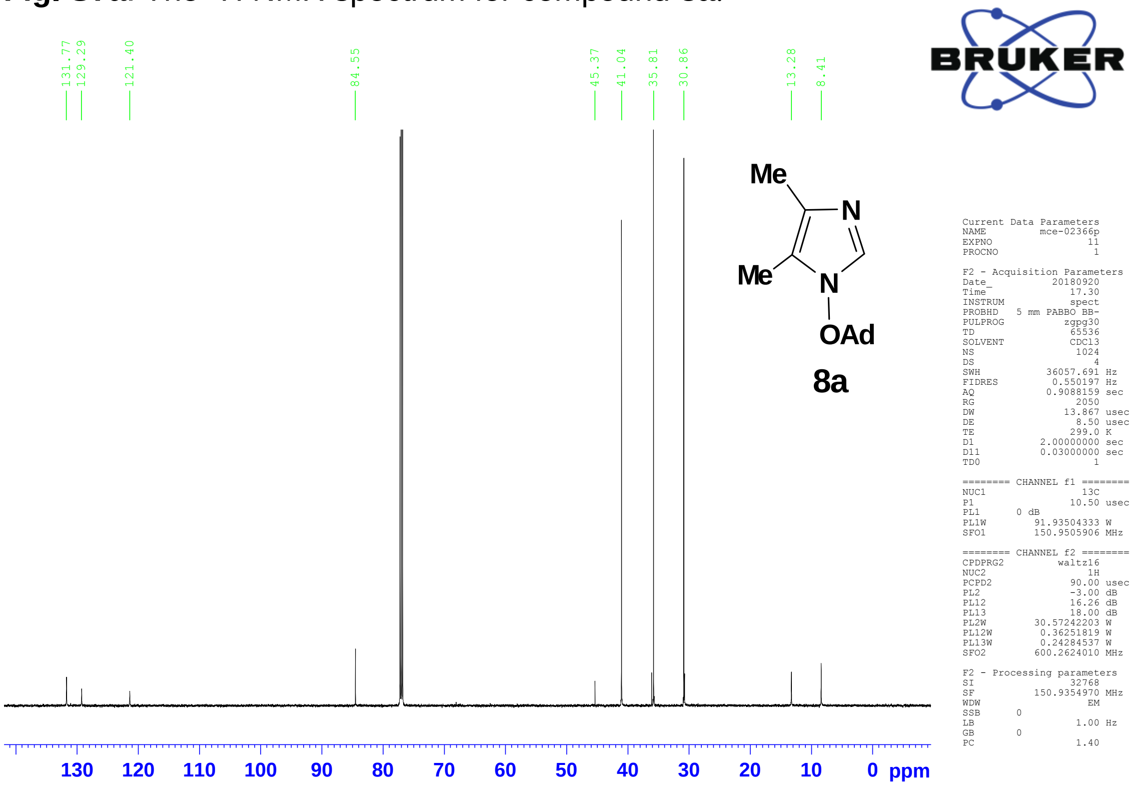

Fig. S7b. The ^{13}C NMR spectrum for compound **8a**.

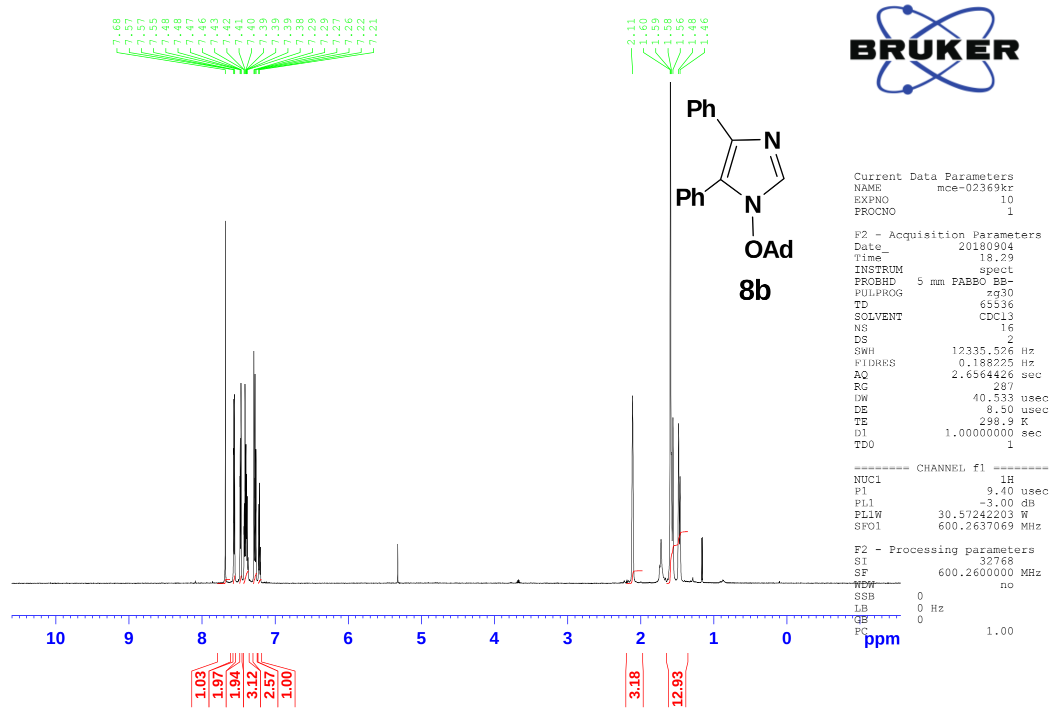

Fig. S8a. The ^{1}H NMR spectrum for compound **8b**.

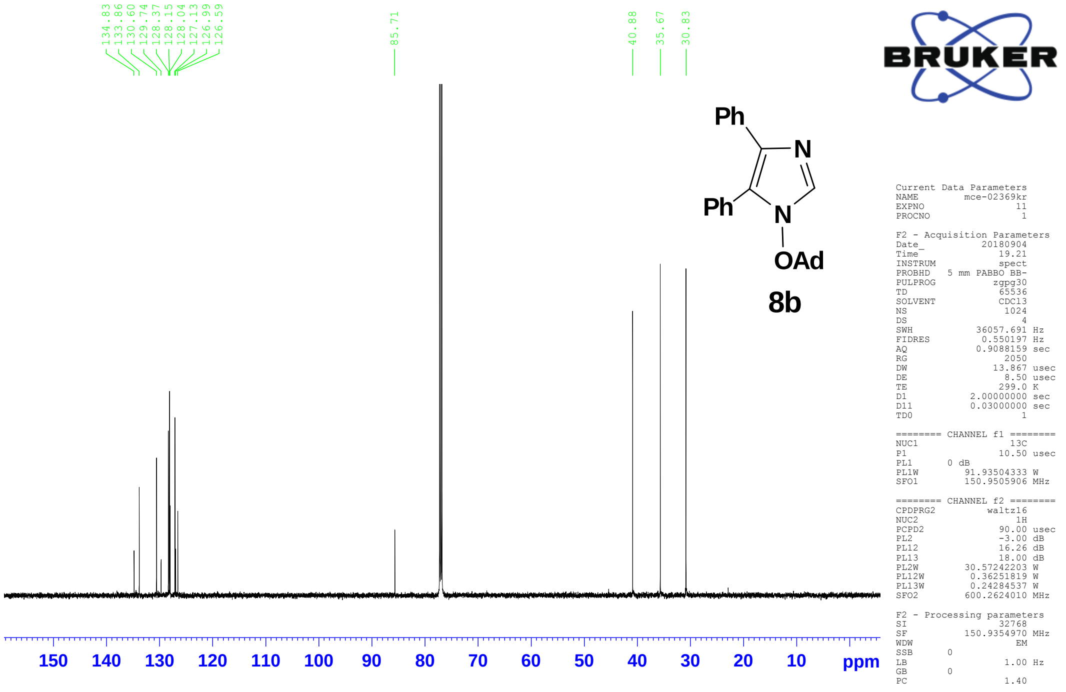

Fig. S8b. The ^{13}C NMR spectrum for compound **8b**.

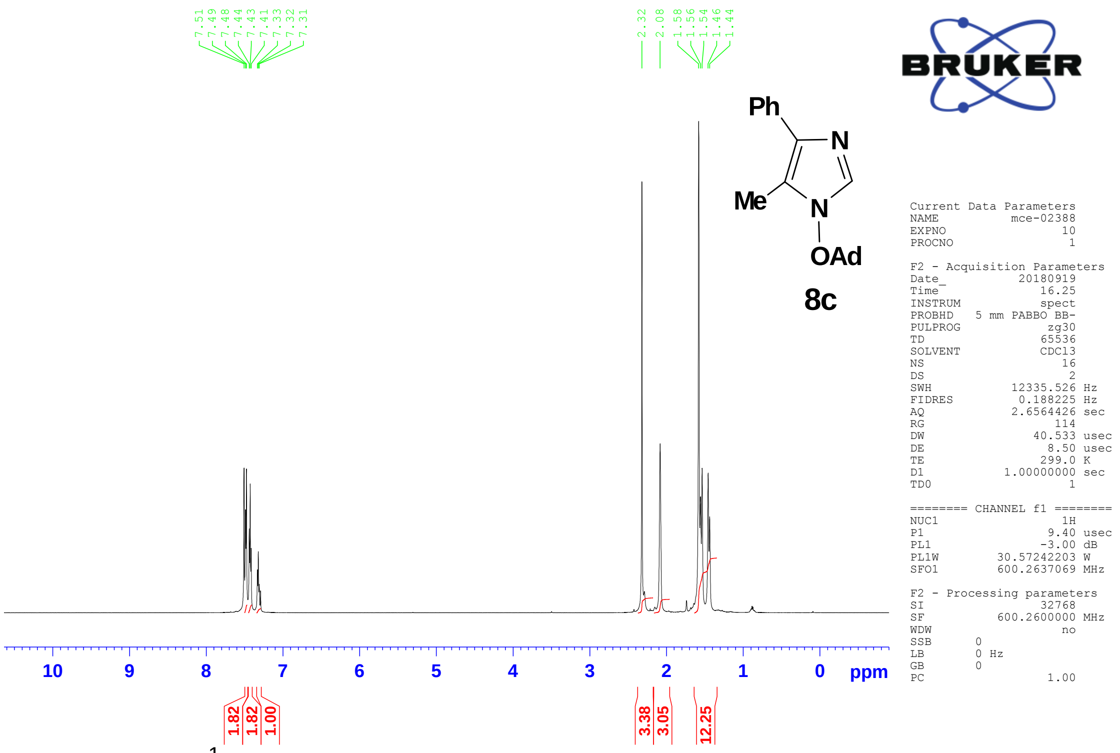

Fig. S9a. The ^{1}H NMR spectrum for compound **8c**.

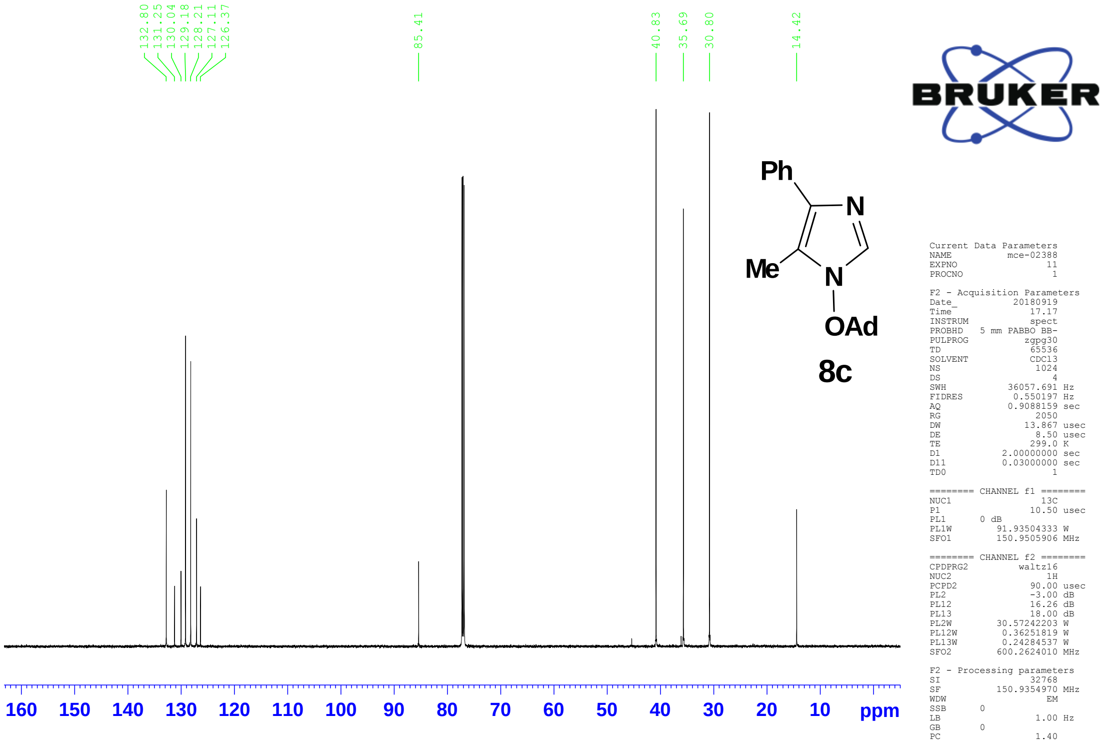

Fig. S9b. The ^{13}C NMR spectrum for compound **8c**.

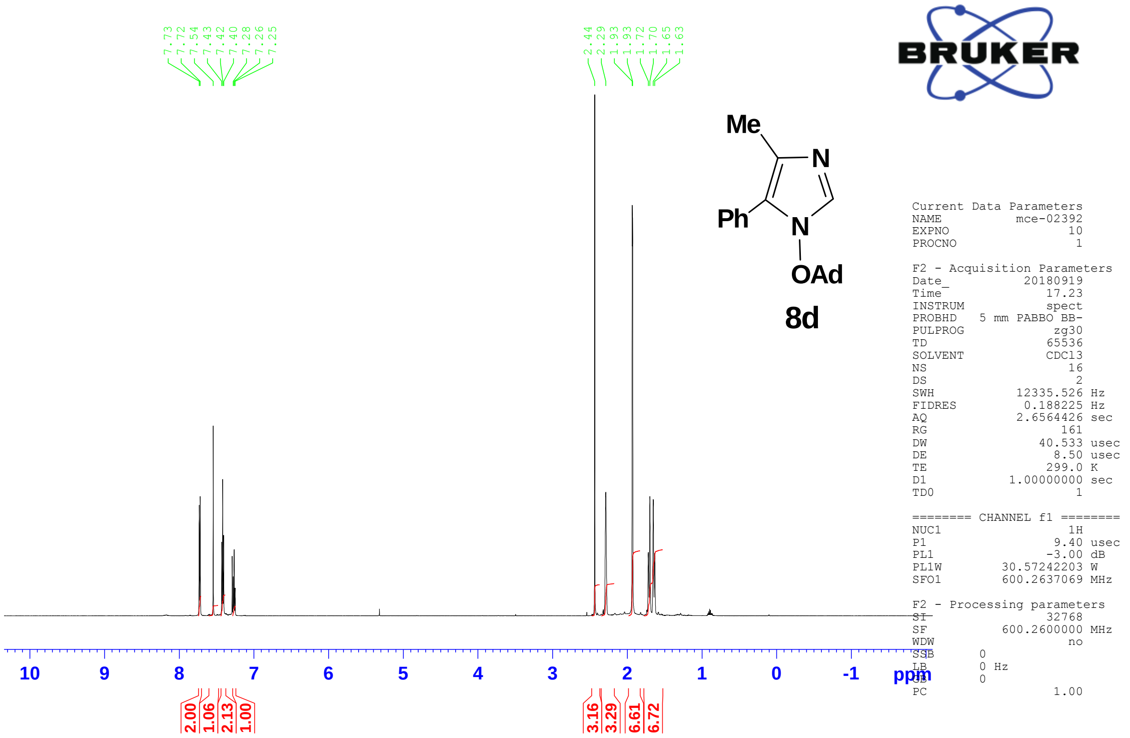

Fig. S10a. The ^{1}H NMR spectrum for compound **8d**.

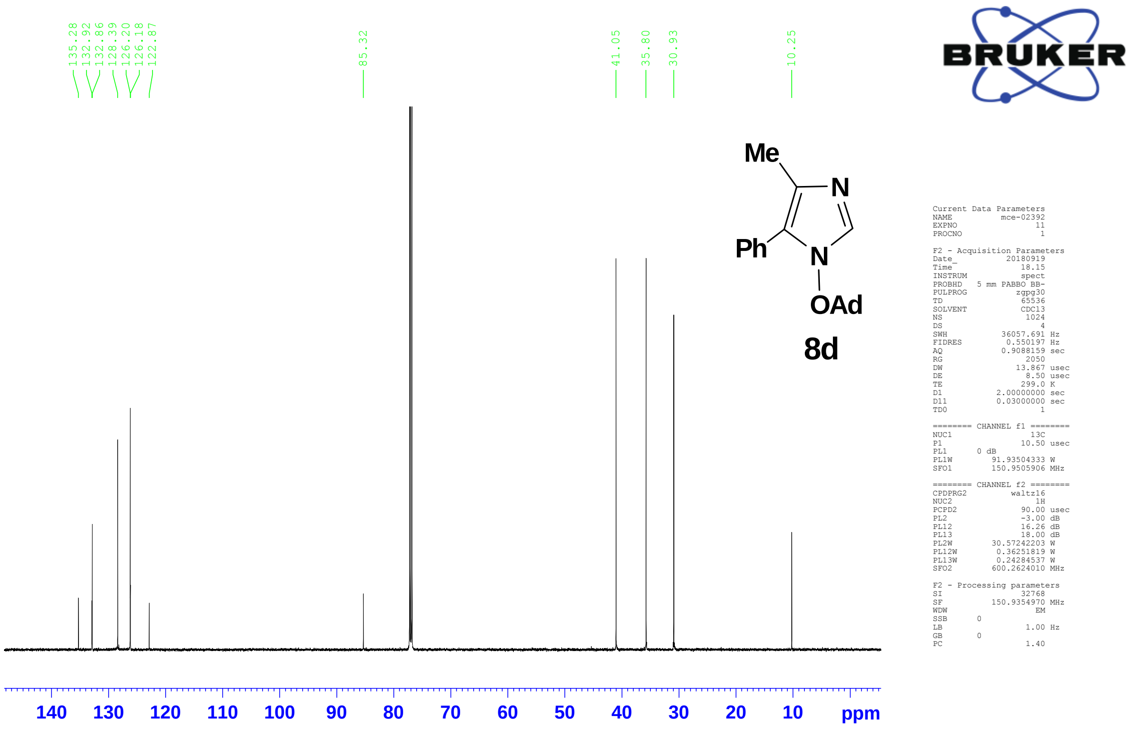

Fig. S10b. The ^{13}C NMR for compound **8d**.

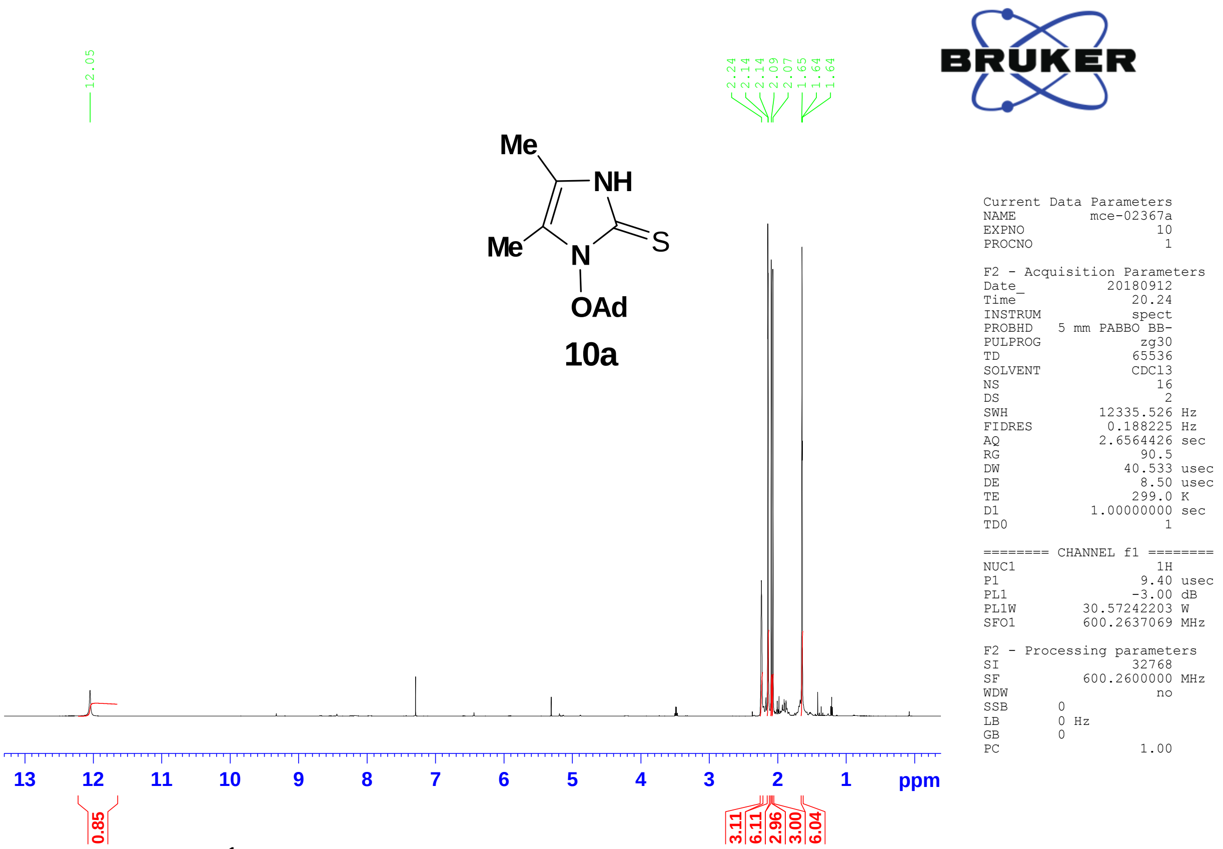

Fig. S11a. The ^{1}H NMR for compound **10a**.

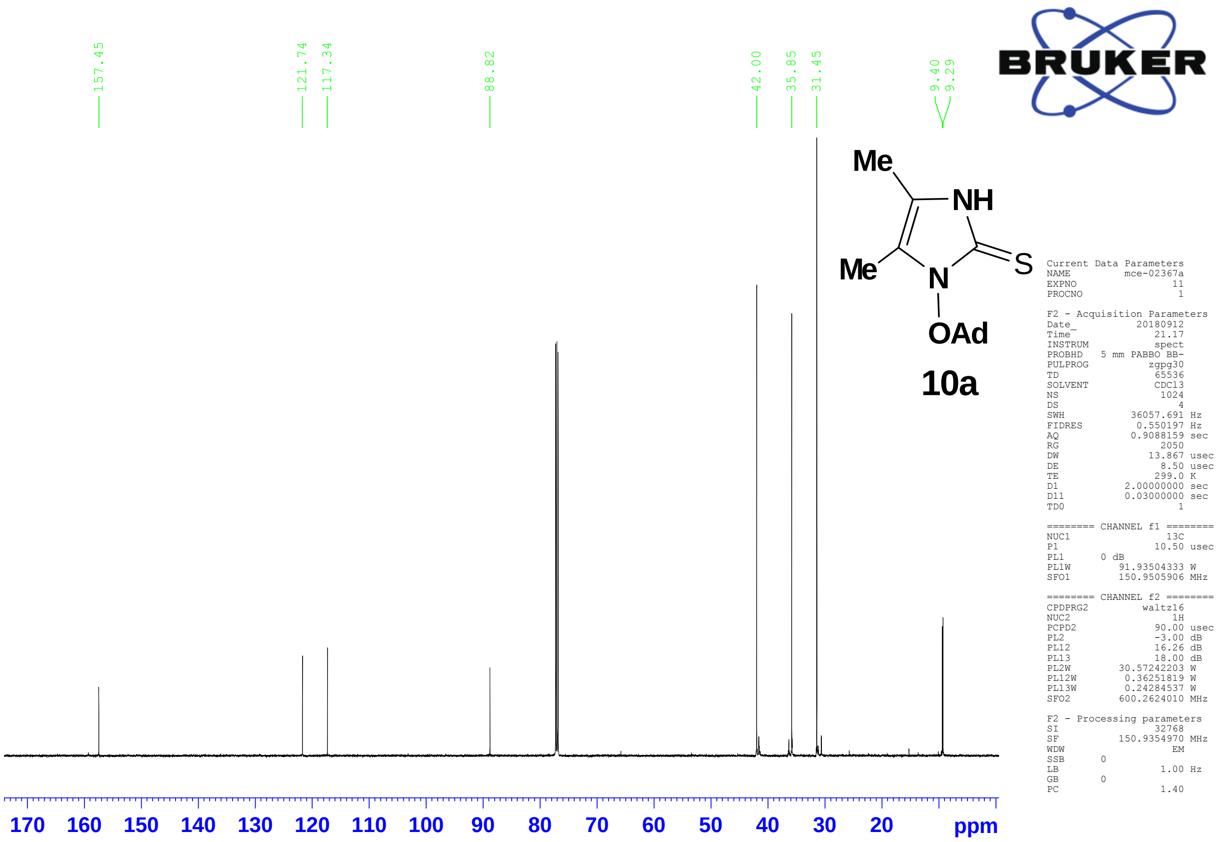

Fig. S11b. The ^{13}C NMR spectrum for compound **10a**.

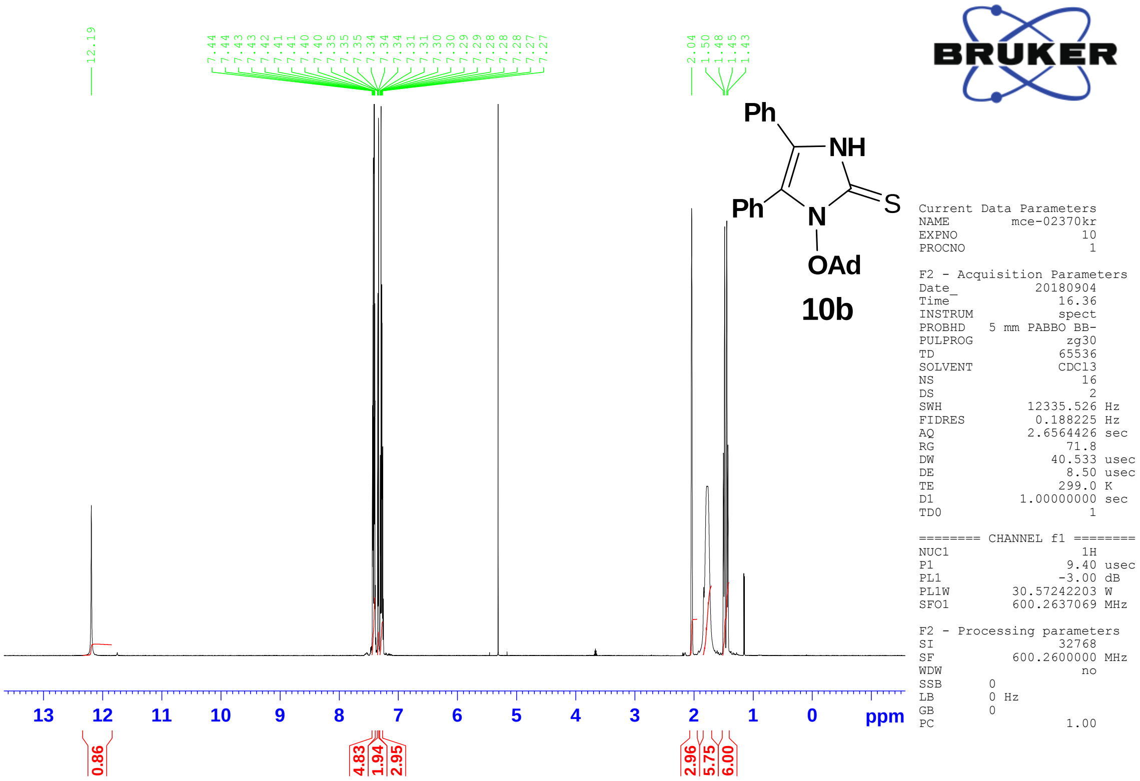

Fig. S12a. The ^{1}H NMR spectrum for compound **10b**.

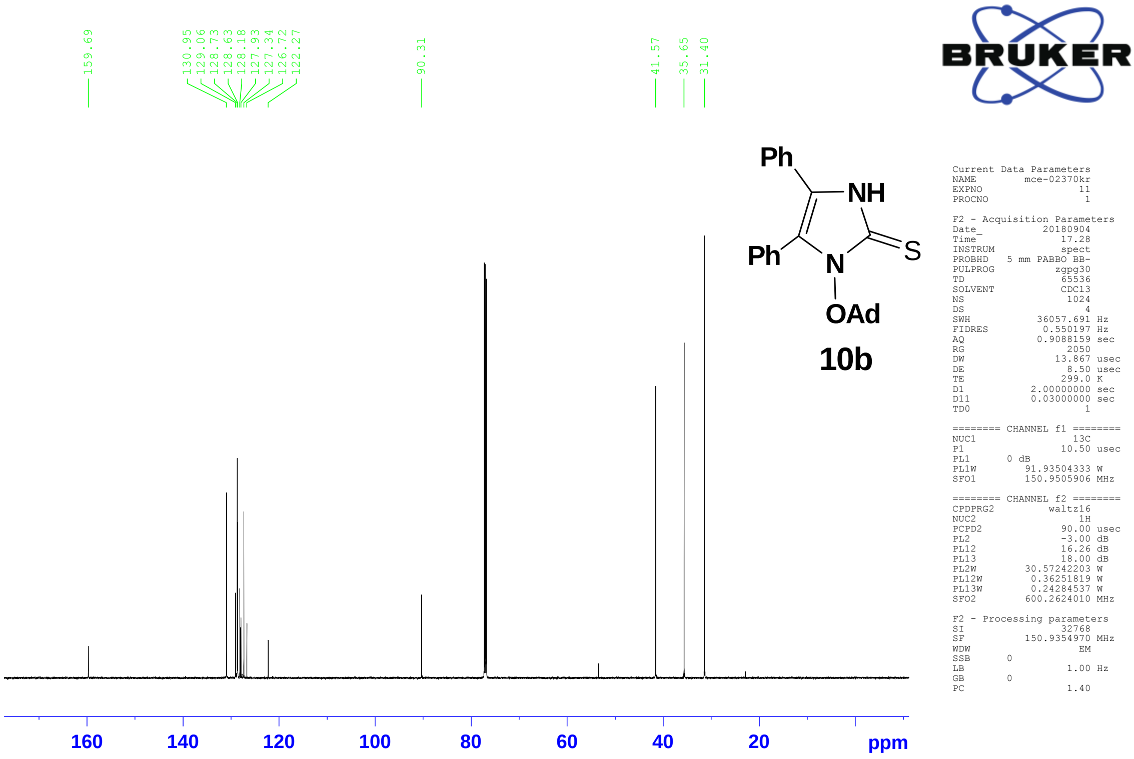

Fig. S12b. The ^{13}C NMR spectrum for compound **10b**.

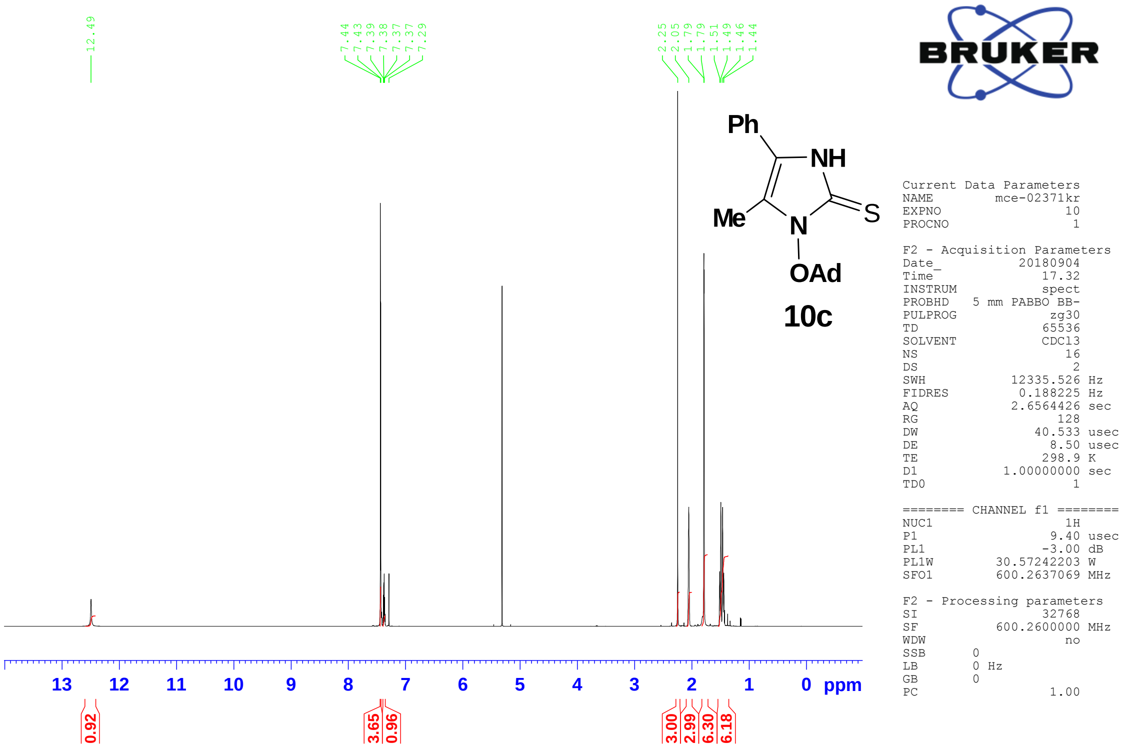

Fig. S13a. The ^{1}H NMR spectrum for compound **10c**.

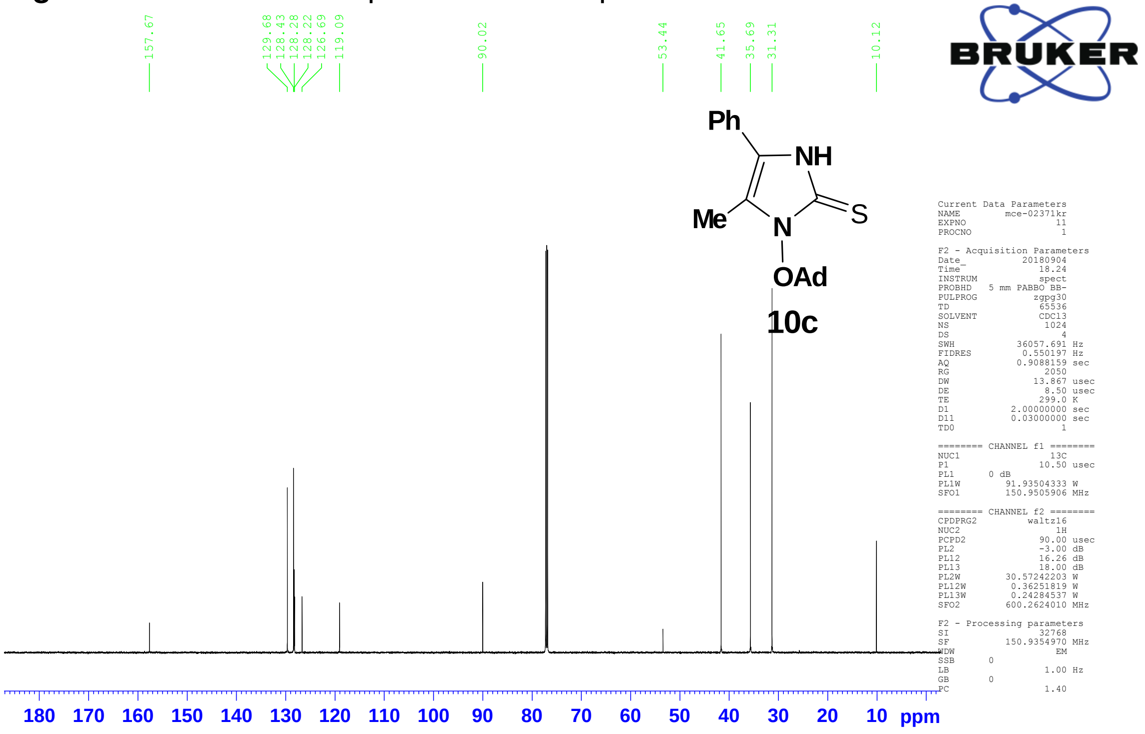

Fig. S13b. The ^{13}C NMR spectrum for compound **10c**.

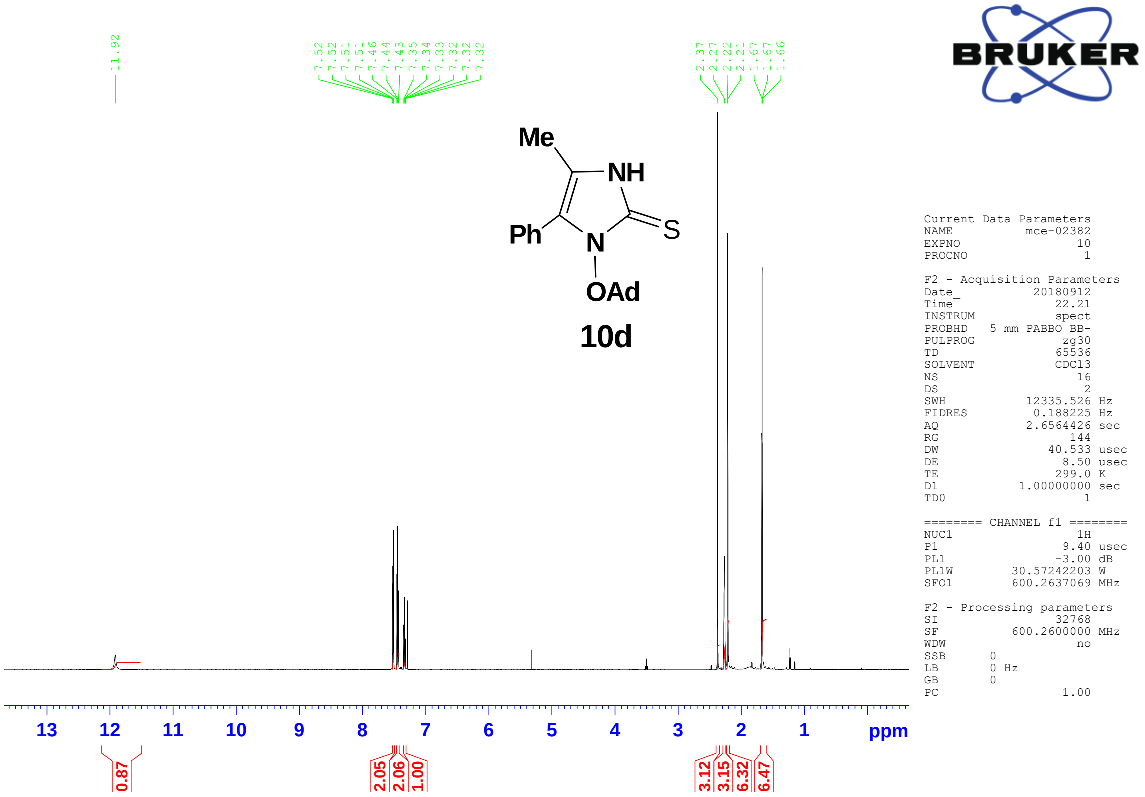

Fig. S14a. The ^{1}H NMR spectrum for compound **10d**.

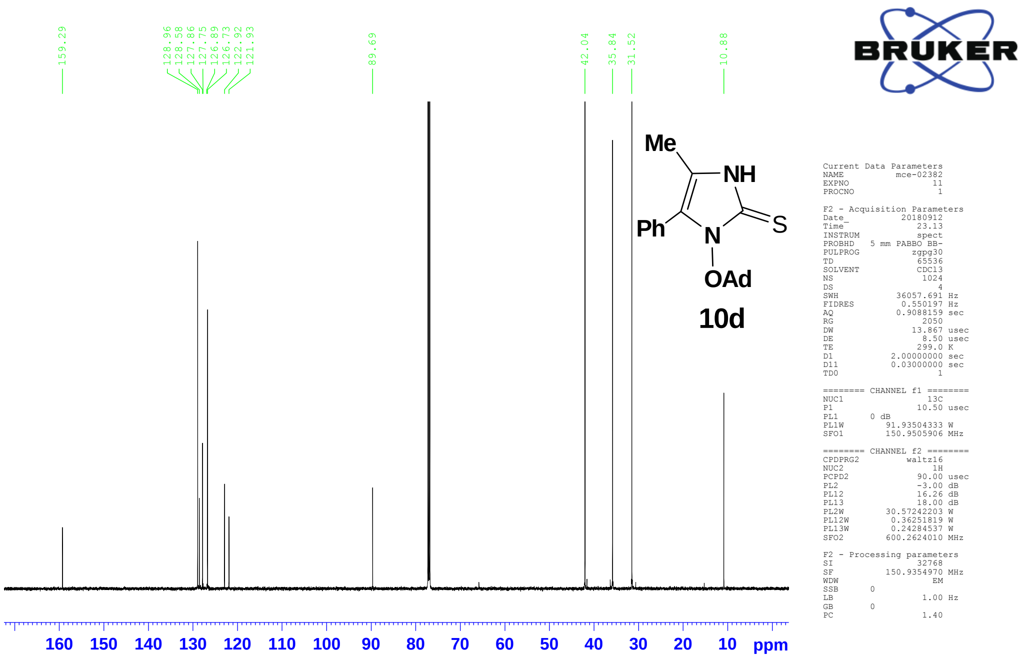

Fig. S14b. The ^{13}C NMR spectrum for compound **10d**.

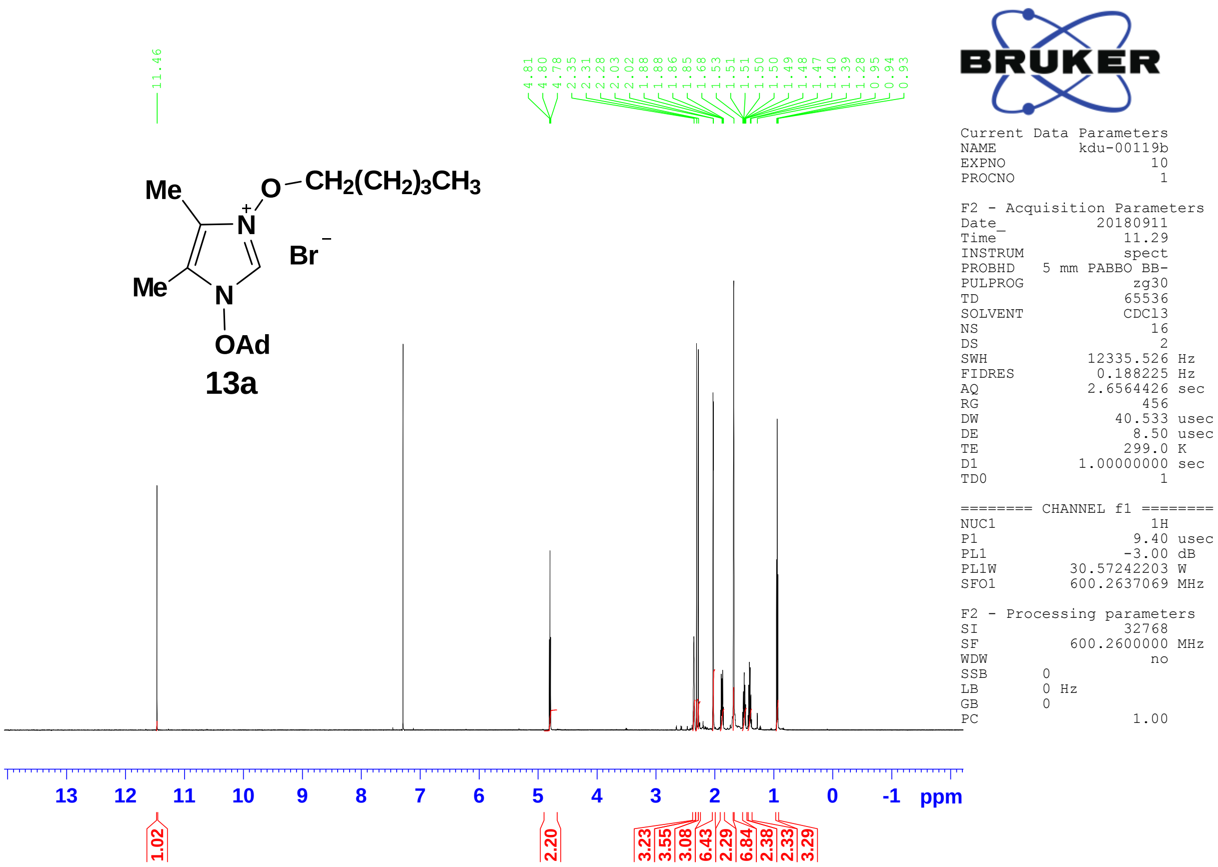

Fig. S15a. The ^{1}H NMR spectrum for compound **13a**.

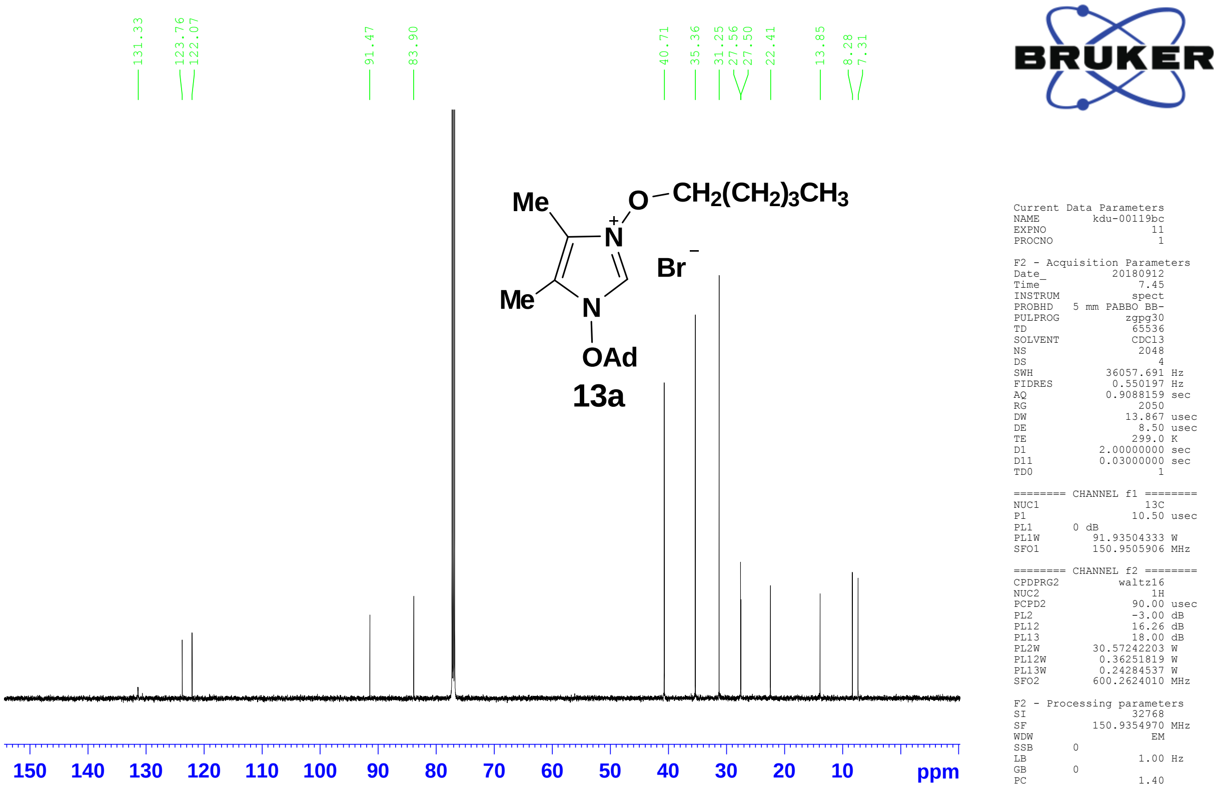

Fig. S15b. The ^{13}C NMR spectrum for compound **13a**.

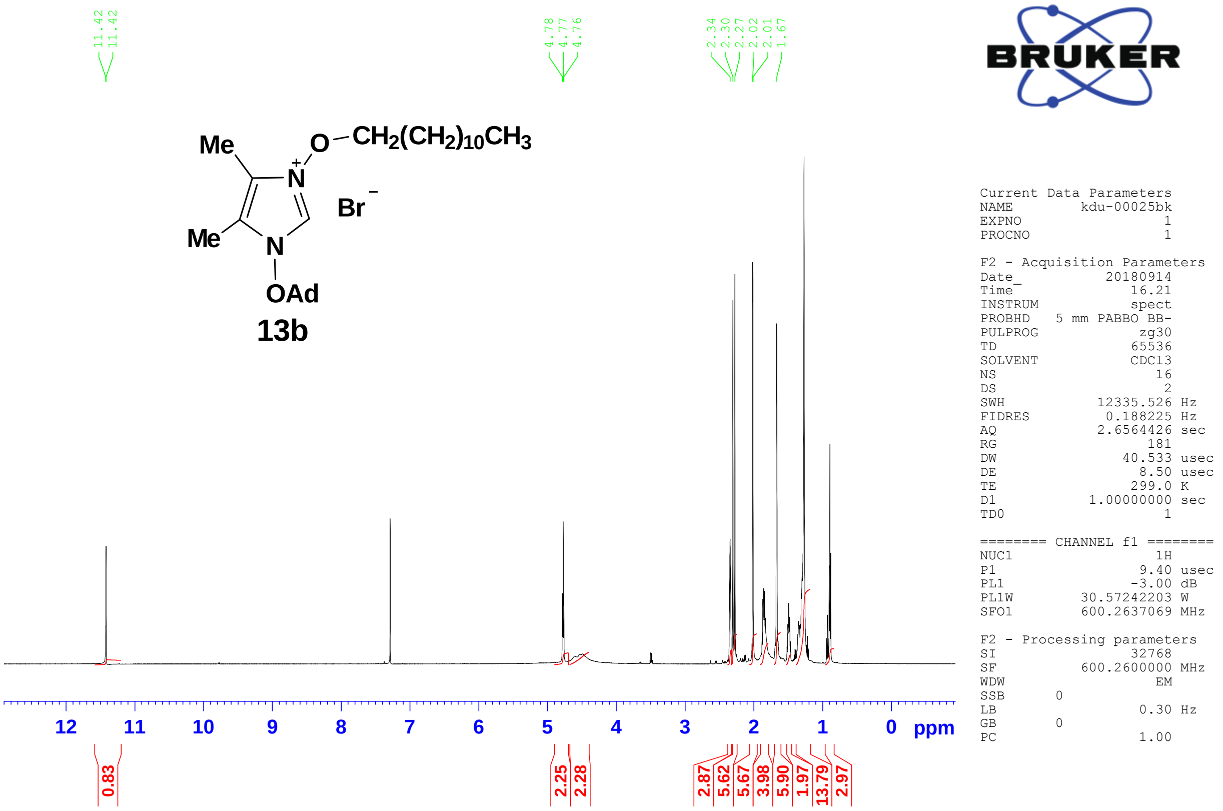

Fig. S16a. The ^{1}H NMR spectrum for compound **13b**.

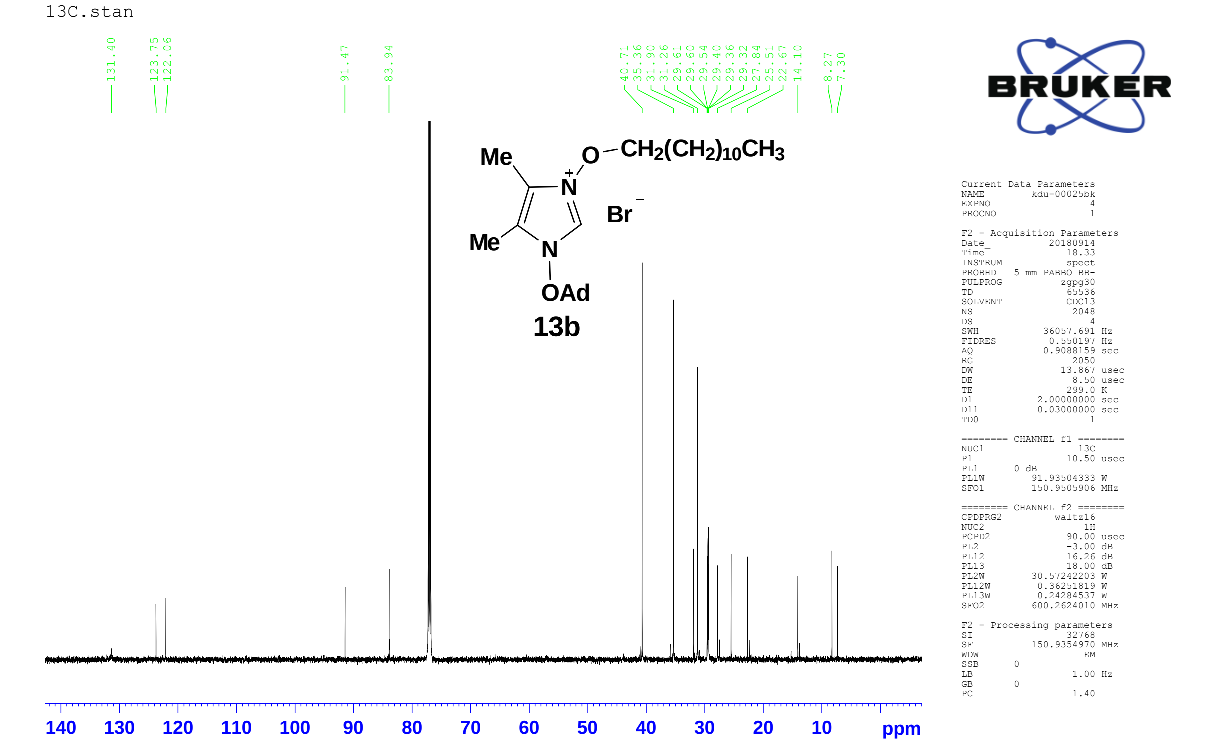

Fig. S16b. The ^{13}C NMR spectrum for compound **13b**.

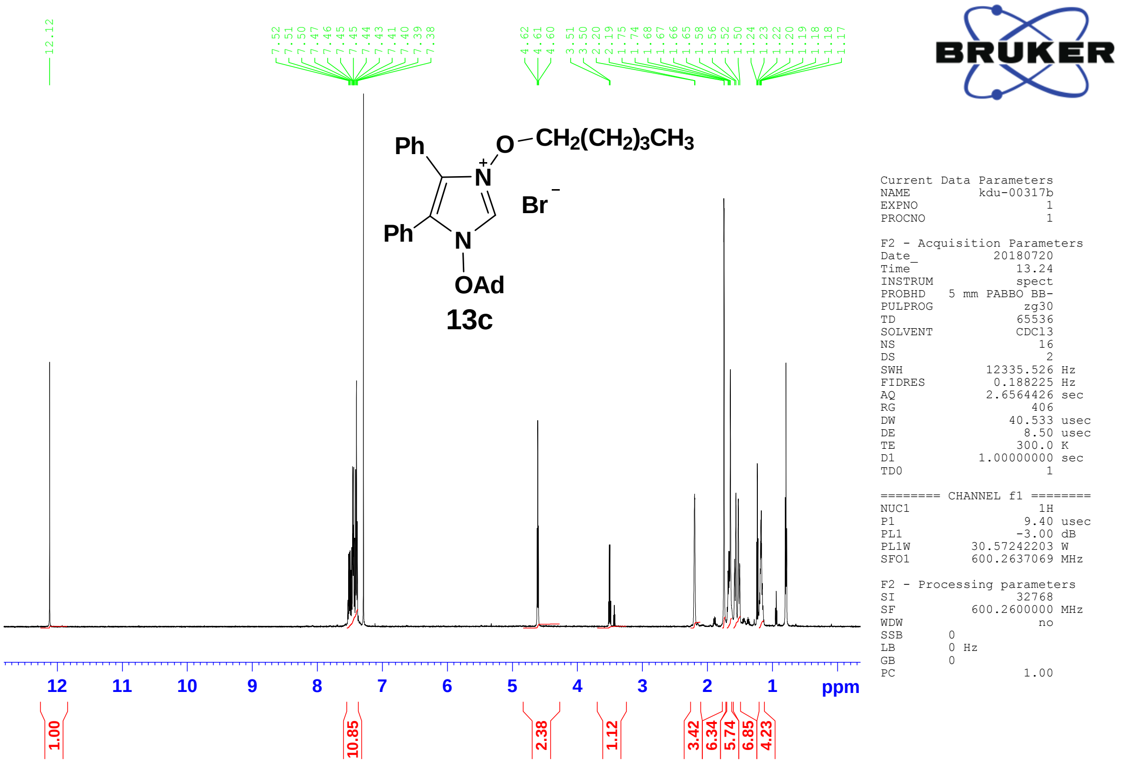

Fig. S17a. The ^{1}H NMR spectrum for compound **13c**.

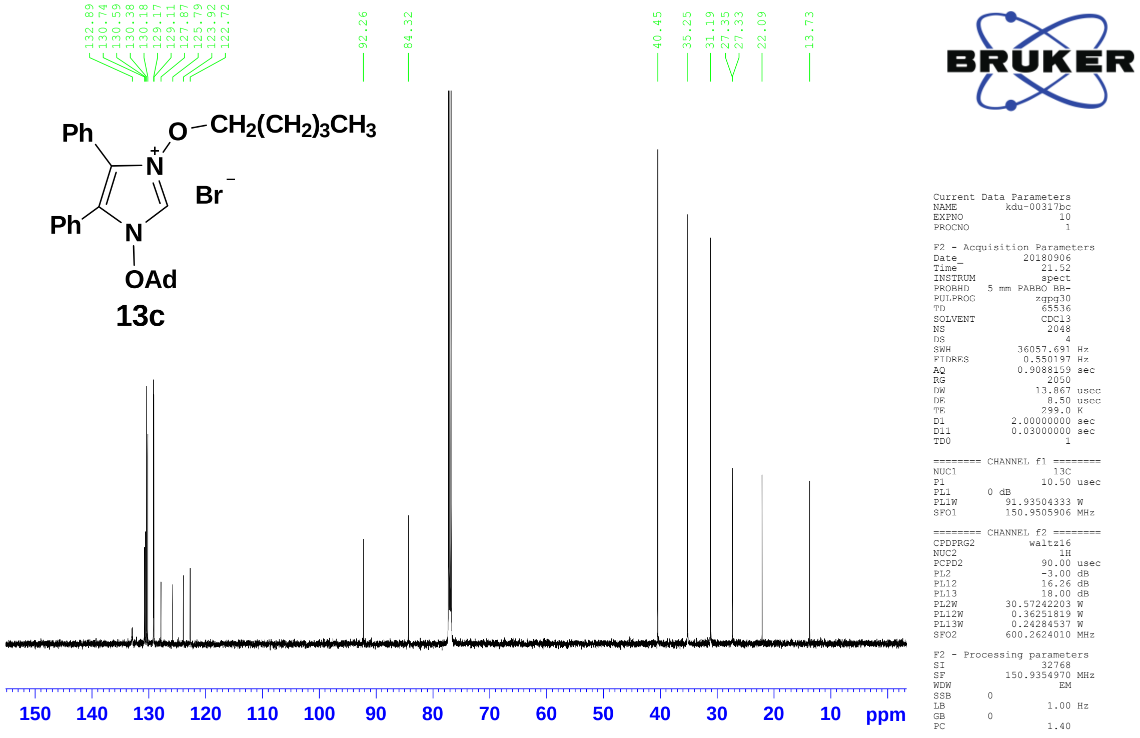

Fig. S17b. The ^{13}C NMR spectrum for compound **13c**.

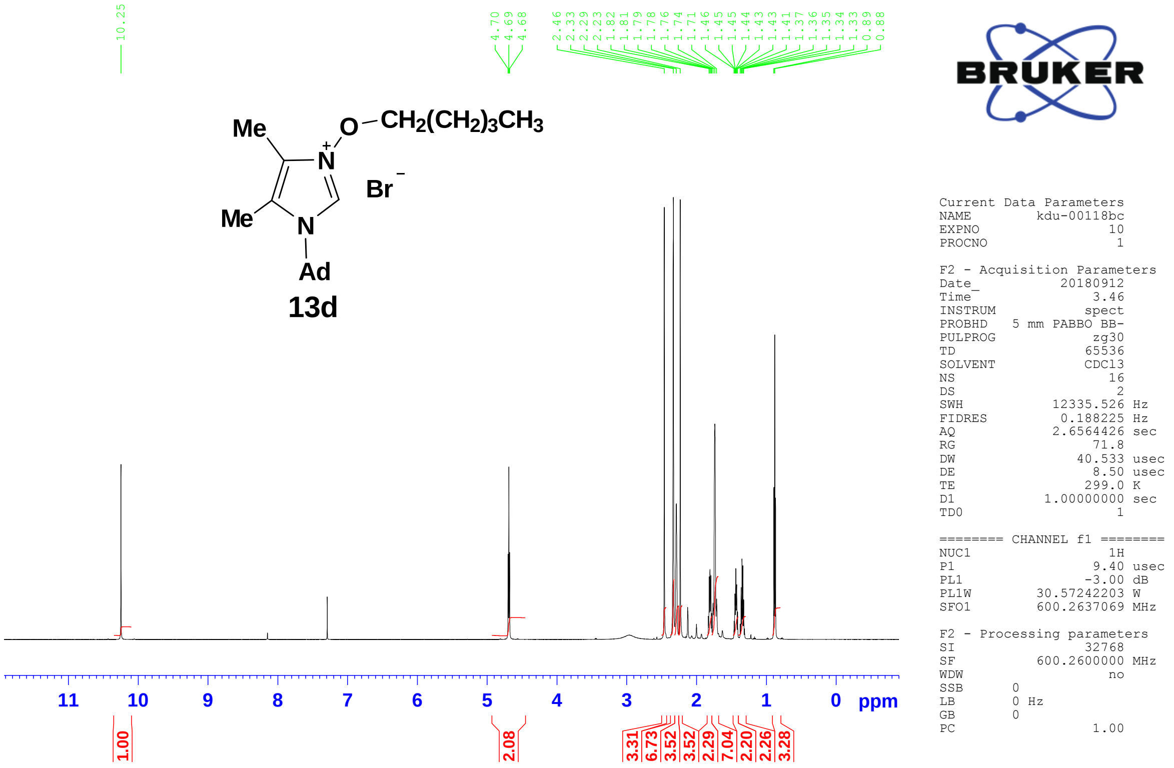

Fig. S18a. The ^{1}H NMR spectrum for compound **13d**.

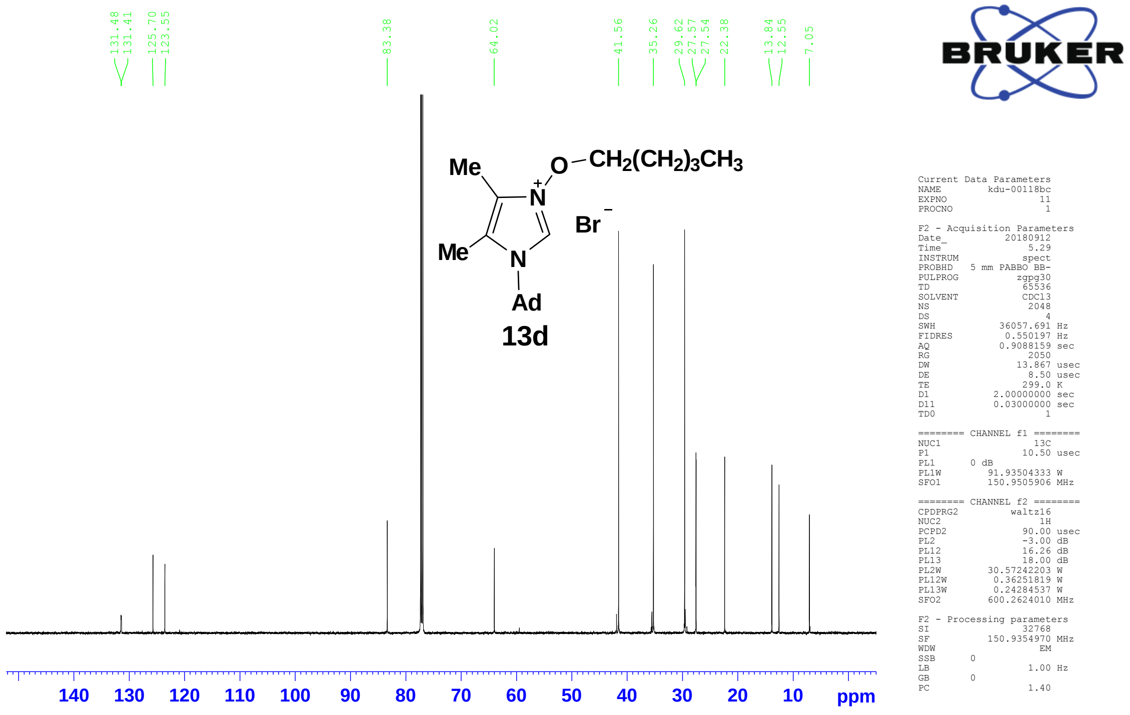

Fig. S18b. The ^{13}C NMR spectrum for compound **13d**.

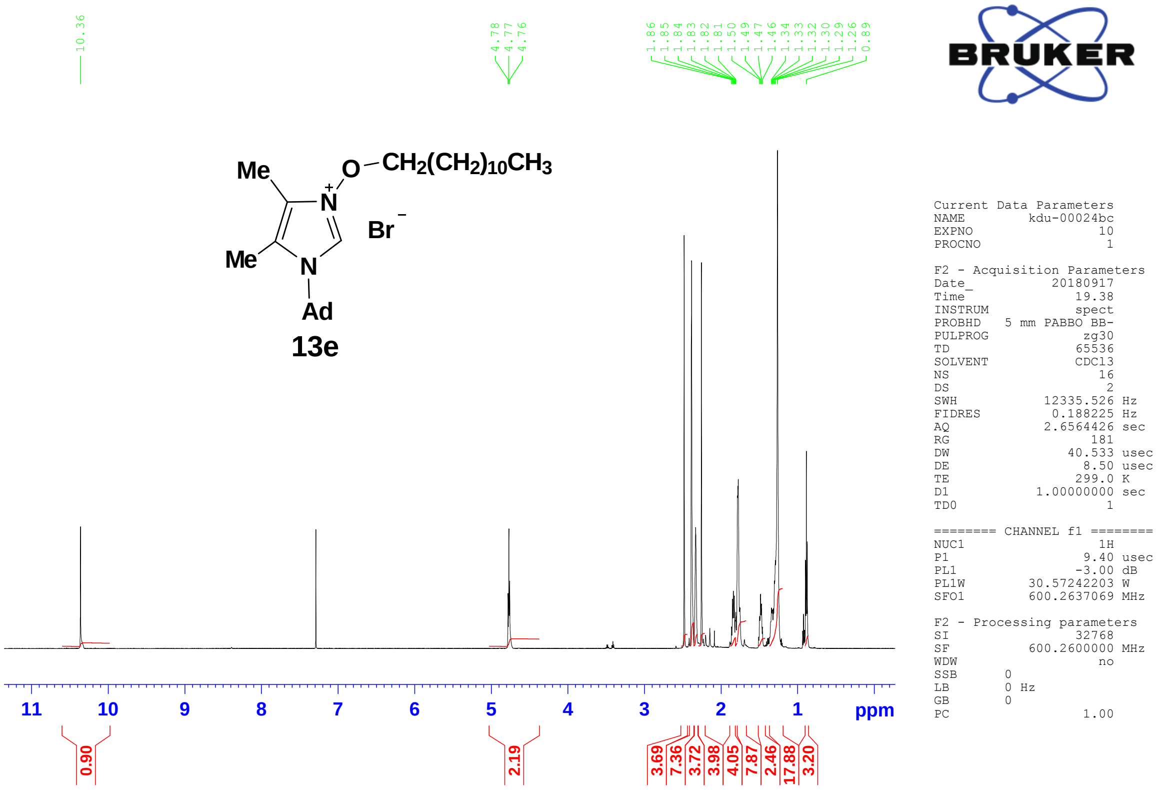

Fig. S19a. The ^{1}H NMR spectrum for compound **13e**.

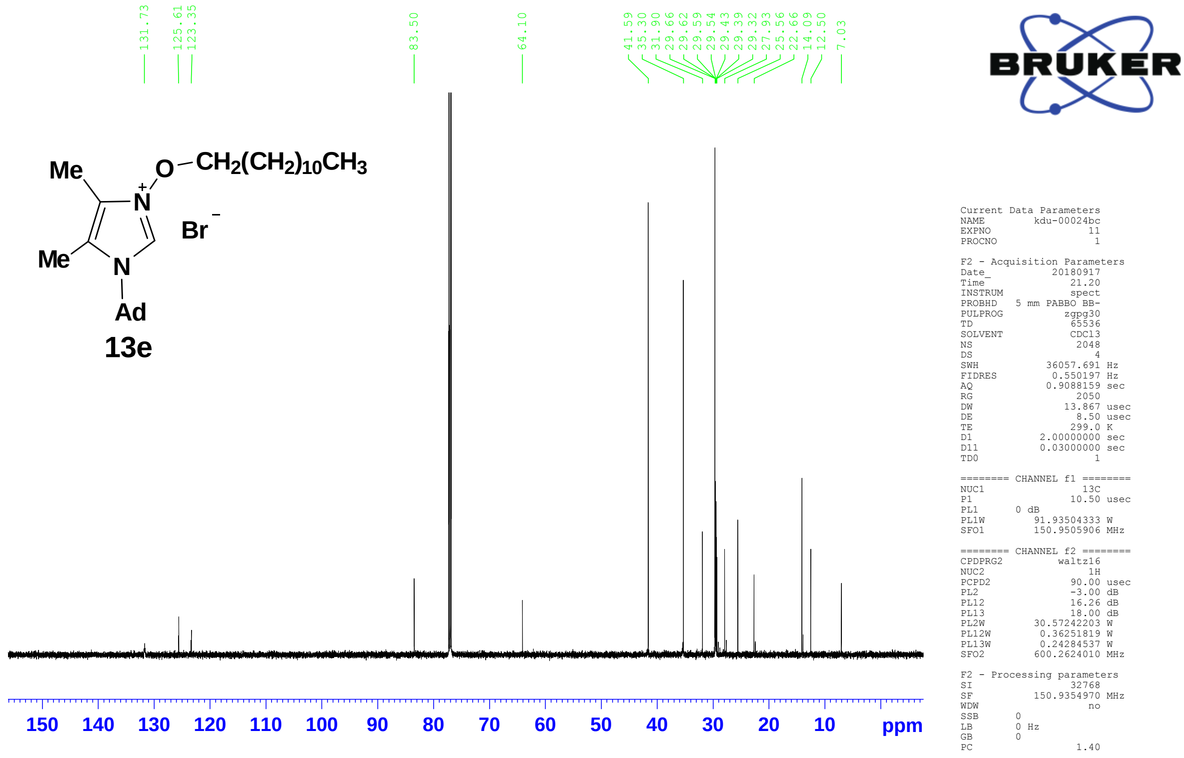

Fig. S19b. The ^{13}C NMR spectrum for compound **13e**.

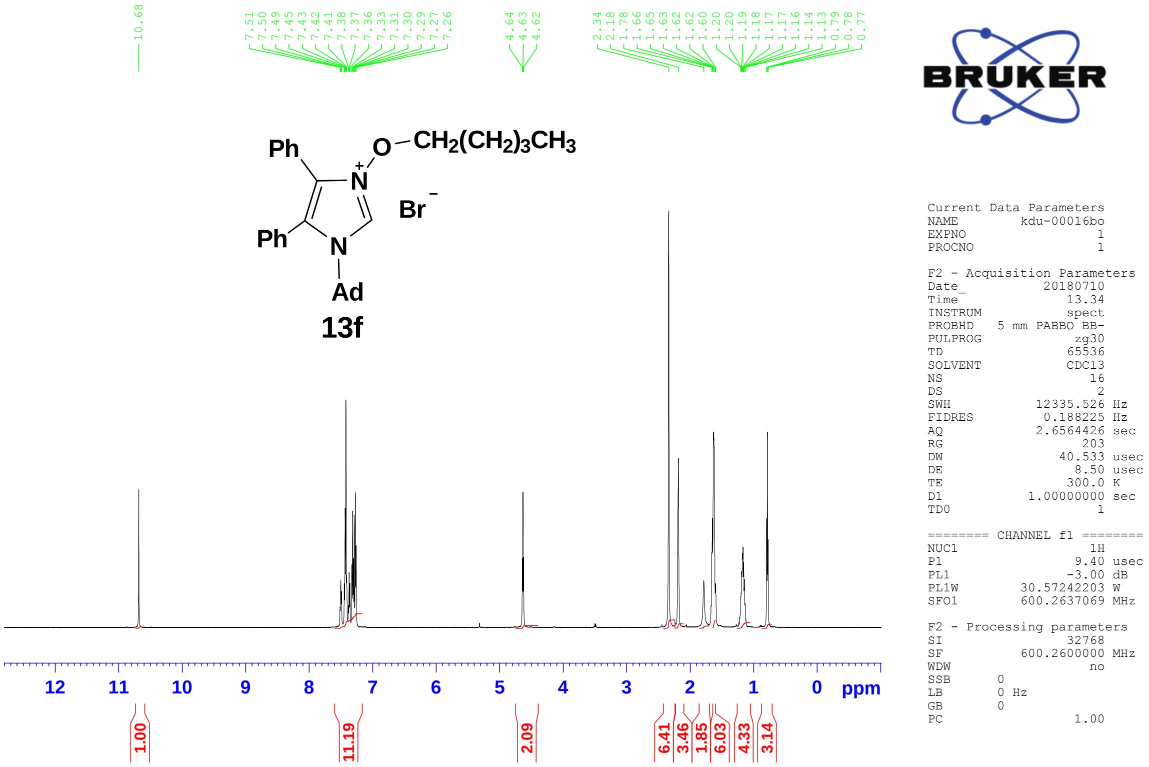

Fig. S20a. The ^{1}H NMR spectrum for compound **13f**.

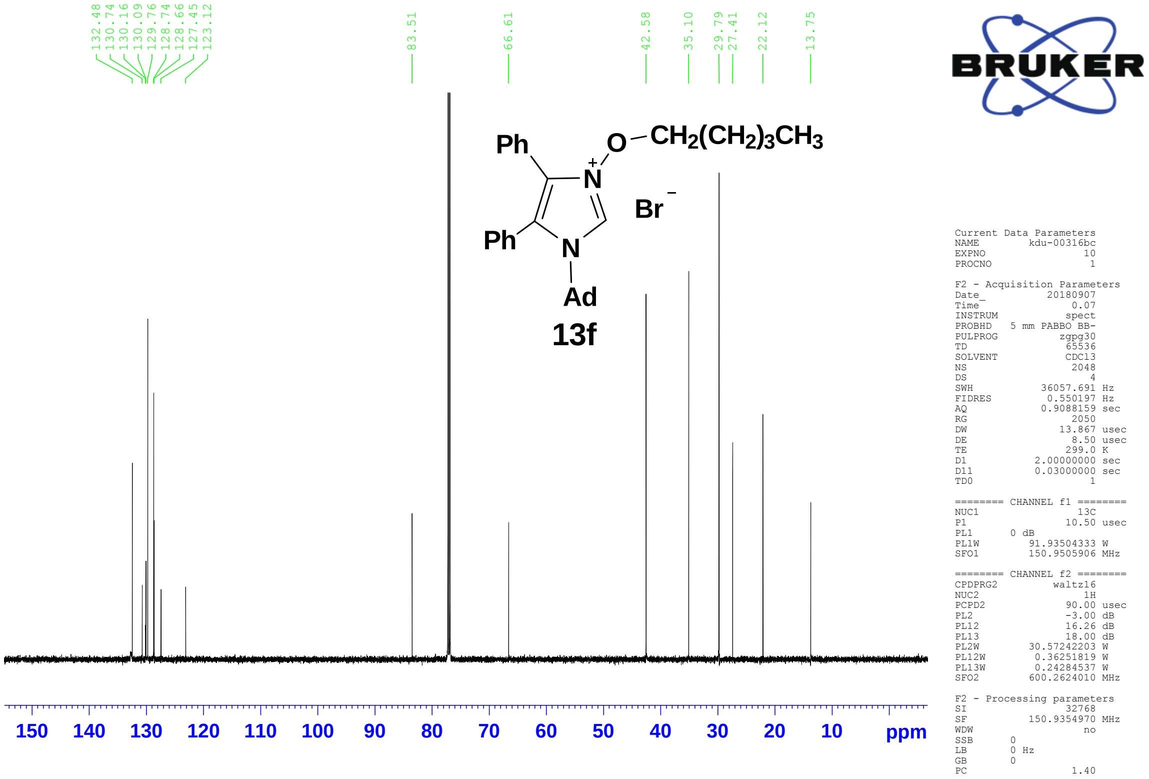

Fig. S20b. The ^{13}C NMR spectrum for compound **13f**.

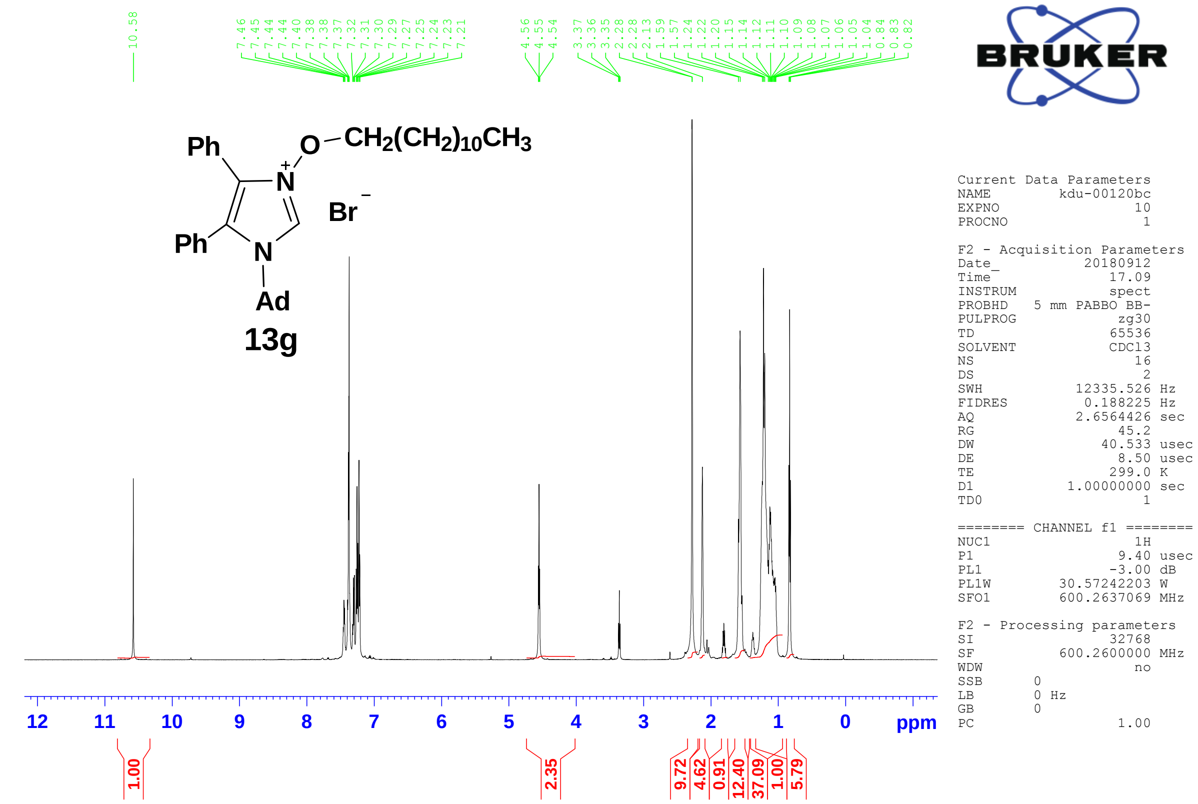

Fig. S21a. The ^{1}H NMR spectrum for compound **13g**.

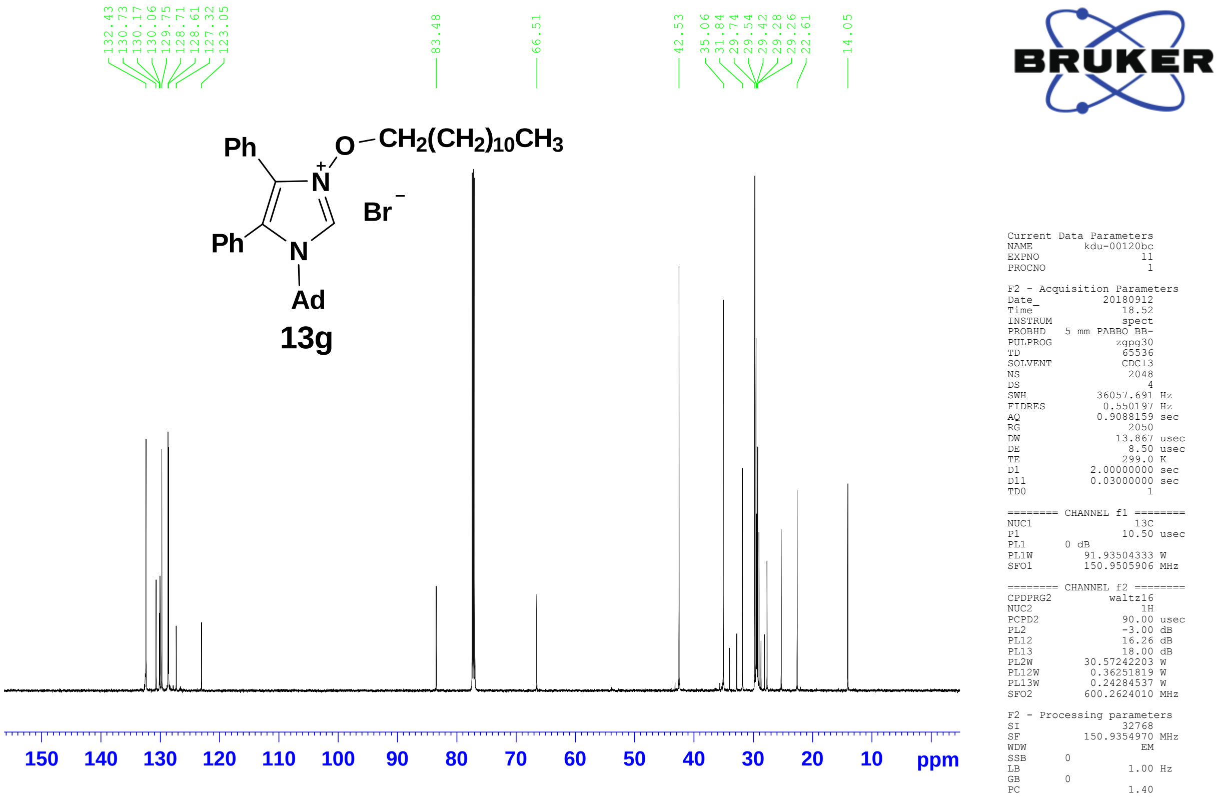

Fig. S21b. The ^{13}C NMR spectrum for compound **13g**.

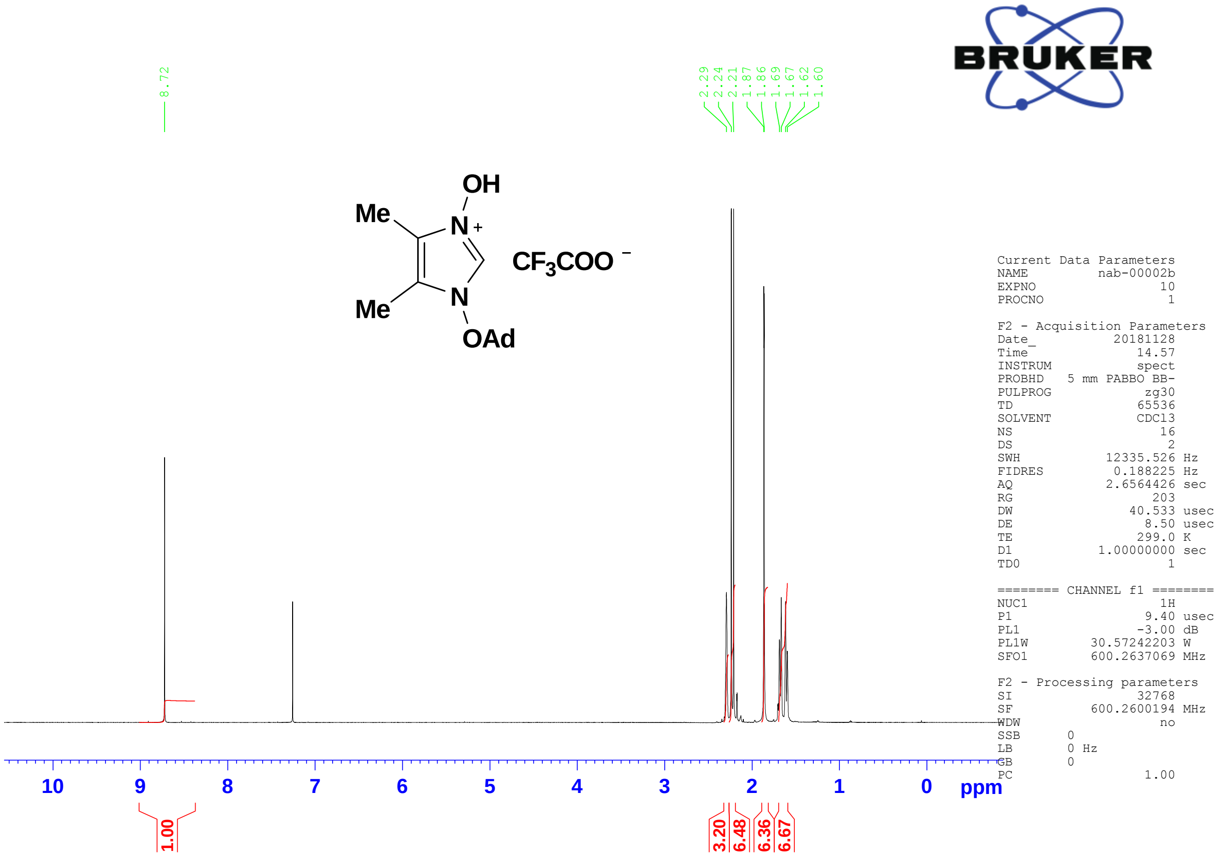

Fig. S22a. The ^{1}H NMR spectrum for compound **14.**

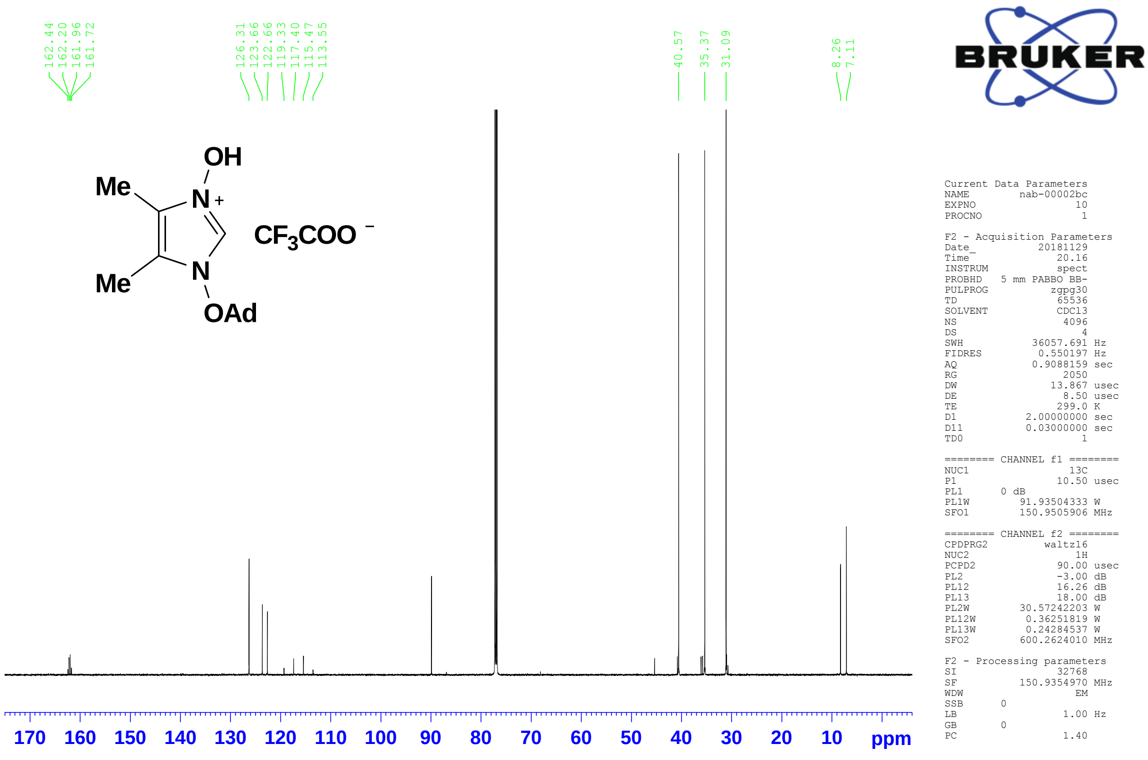

Fig. S22b. The ^{13}C NMR spectrum for compound **14.**

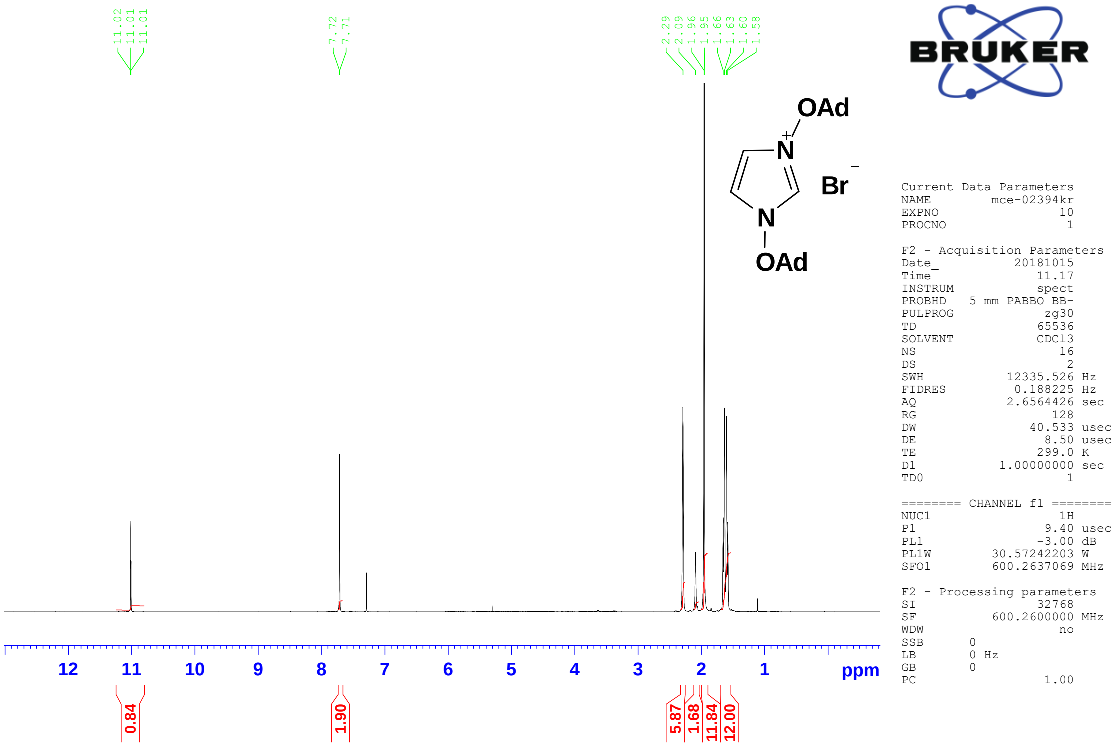

Fig. S23a. The ^{1}H NMR spectrum for compound **15**.

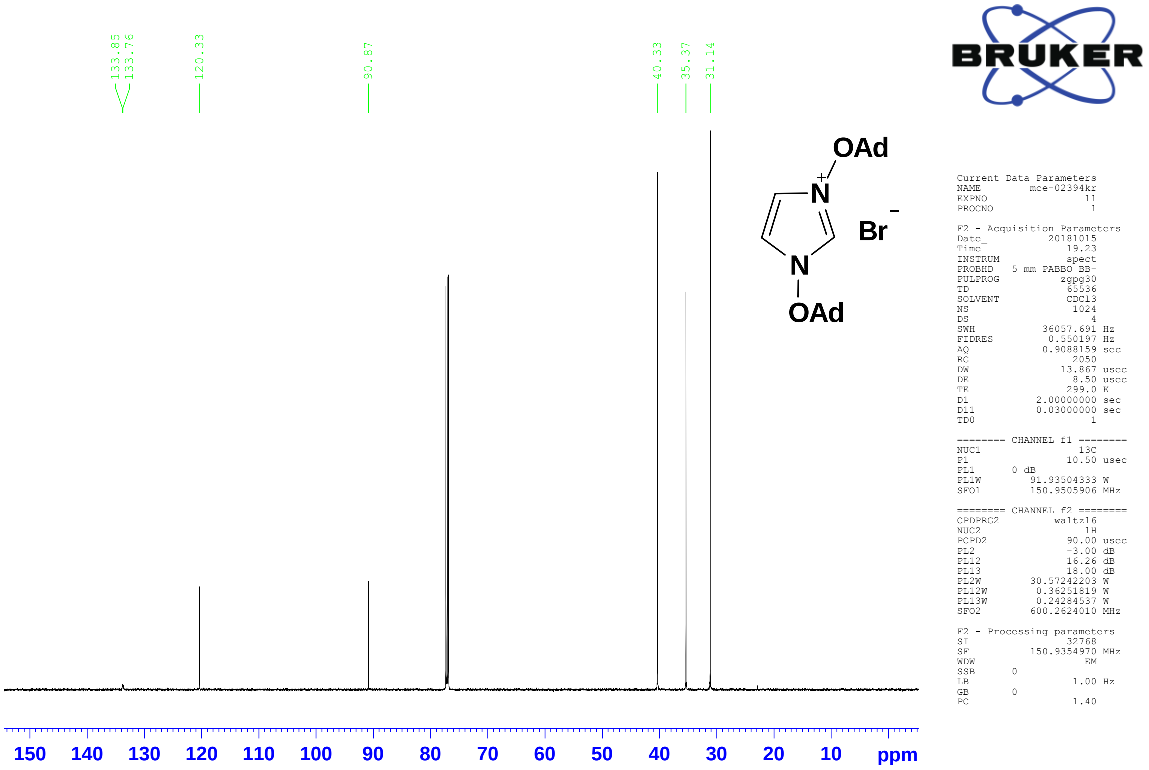

Fig. S23b. The ^{13}C NMR spectrum for compound **15**.
